# A cell‐penetrating peptide exerts therapeutic effects against ischemic stroke by mediating the lysosomal degradation of sirtuin 5

**DOI:** 10.1002/mco2.436

**Published:** 2023-12-13

**Authors:** Qian Xia, Xue Zhang, Gaofeng Zhan, Lu Zheng, Meng Mao, Yin Zhao, Yilin Zhao, Xing Li

**Affiliations:** ^1^ Department of Anesthesiology Hubei Key Laboratory of Geriatric Anesthesia and Perioperative Brain Health, and Wuhan Clinical Research Center for Geriatric Anesthesia Tongji Hospital Tongji Medical College Huazhong University of Science and Technology Wuhan China; ^2^ Department of Transfusion The First Affiliated Hospital of Zhengzhou University Zhengzhou China; ^3^ Department of Anesthesiology and Perioperative Medicine Zhengzhou Central Hospital Affiliated to Zhengzhou University Zhengzhou China; ^4^ Department of Ophthalmology Tongji Hospital Tongji Medical College Huazhong University of Science and Technology Wuhan China

**Keywords:** annexin‐A1, cell‐penetrating peptide, ischemic stroke, microglia, neuroinflammation, sirtuin 5

## Abstract

Stroke is a major public health concern worldwide. The lack of effective therapies heightens the need for new therapeutic agents. Previous study identified sirtuin 5 (SIRT5) as a positive regulator of microglia‐induced excessive neuroinflammation following ischemic stroke. Interventions targeting SIRT5 should therefore alleviate neuroinflammation and protect against ischemic stroke. Here, we synthesized a membrane‐permeable peptide specifically bound to SIRT5 through a chaperone‐mediated autophagy targeting motif (Tat‐SIRT5‐CTM) and examined its therapeutic effect in vitro and in vivo. First, in primary microglia, Tat‐SIRT5‐CTM suppressed the binding of SIRT5 with annexin‐A1 (ANXA1), leading to SIRT5 degradation and thus inhibition of SIRT5‐mediated desuccinylation of ANXA1, followed by increased membrane accumulation and secretion of ANXA1. These changes, in turn, alleviated microglia‐induced neuroinflammation. Moreover, following intravenous injection, Tat‐SIRT5‐CTM could efficiently pass through the blood‒brain barrier. Importantly, systemic administration of Tat‐SIRT5‐CTM reduced the brain infarct area and neuronal loss, mitigated neurological deficit scores, and improved long‐term neurological functions in a mouse model of ischemic stroke. Furthermore, no toxicity was observed when high doses Tat‐SIRT5‐CTM were injected into nonischemic mice. Collectively, our study reveals the promising efficacy of the peptide‐directed lysosomal degradation of SIRT5 and suggests it as an effective therapeutic approach for the treatment of ischemic stroke.

## Graphical abstract

Xia et al. synthesize a cell‐penetrating peptide Tat‐SIRT5‐CTM and reveal that this peptide specifically blocked the interaction of SIRT5 with ANXA1, led to SIRT5 degradation and thereby inhibiting SIRT5‐mediated desuccinylation of ANXA1, which, in turn, alleviated microglia‐induced neuroinflammation, and eventually protected neurons against ischemic stroke. Thus, the fusion peptide Tat‐SIRT5‐CTM may be a promising therapeutic candidate for the treatment of ischemic stroke.

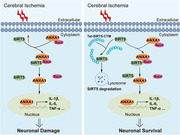

## 1 BACKGROUND

Ischemic stroke remains a leading cause of disability and death worldwide.^1^, ^2^ Without effective interventions, ischemic lesions become irreversible in the first few hours after blood flow is occluded. The current ischemic stroke treatments are limited to rapid thrombolysis or endovascular thrombus removal to restore brain perfusion.^3^ Intravenous recombinant tissue plasminogen activator (tPA) remains the only specific pharmacological therapy for cerebral ischemia, but the narrow time window (within 4.5 h) of thrombolytic therapy and risk of hemorrhagic transformation limit its use.^4^, ^5^, ^6^ Thus, new therapeutic approaches are urgently needed. Numerous investigations have shown that microglia‐induced neuroinflammation is a critical contributor to the pathogenesis of cerebral ischemia.^7^, ^8^, ^9^ Therefore, studying targets related to the inhibition of neuroinflammation may aid the development of treatments for ischemic stroke.

The sirtuin (SIRT) is a family of seven proteins that have different biological functions, such as aging, energy control, apoptosis, and stress resistance.^10^, ^11^ Most SIRTs exert their effects through protein deacetylation. Unlike other SIRTs, SIRT5 possesses weak deacetylase activity but strong protein desuccinylase activity.^12^, ^13^, ^14^ We previously reported that SIRT5 interacts with and desuccinylates annexin‐A1 (ANXA1) at lysine residue 166, promoting ANXA1 nuclear translocation and suppressing its membrane accumulation and secretion by microglia, which mediates the inflammatory response and eventually aggravates ischemic stroke injury.^15^ In contrast, targeting abnormal SIRT5 has a neuroprotective effect against ischemic stroke. Actually, knockdown of SIRT5 using genetic manipulations, such as stereotaxic injection of antisense oligonucleotide or short hairpin RNA (shRNA), has been demonstrated to protect against neuronal damage in focal brain ischemia model mice.^15^ However, the clinical applications of these methods into effective stroke treatment have been hampered by their limited ability to cross the blood‒brain barrier (BBB). Therefore, a new SIRT5 knockdown approach involving peripheral delivery of an agent that can efficiently cross the BBB might be a promising treatment for ischemic stroke.

Previous studies have identified a powerful and convenient research method to manipulate endogenous protein levels, a method based on membrane‐permeable targeted peptides that rapidly and reversibly knock down endogenous proteins in vitro and in vivo via chaperonin‐mediated autophagy (CMA).^16^ During CMA, heat‐shock cognate protein 70 (HSC70) recognizes soluble proteins that contain the CMA‐targeting motif (CTM) sequence.^17^ The HSC70–substrate complex then binds to lysosome‐associated membrane protein 2 (LAMP2), which transports the substrate to the lysosome lumen.^18^ In this way, pathogenic or misfolded proteins might be degraded by chimera peptides that contain both the CTM motif and a targeting protein‐binding sequence. To enhance their ability to pass through the plasma membrane, these chimeric peptides can be conjugated to the cell‐penetrating peptide trans‐activator of transcription (Tat), which can allow peptides to pass through both the BBB and the plasma membrane after systemic administration.^19^ Therefore, three functional domains comprise CMA‐based degraders: a Tat sequence, a targeting protein‐binding sequence, and a CTM sequence.^16^ CMA‐based degraders first enter the cell and then attach to the target protein via the binding motif and deliver it to the lysosome for degradation. This method has been shown to reduce cyclin‐dependent kinase 5 (CDK5), huntingtin protein, postsynaptic density protein‐95 (PSD‐95), death‐associated protein kinase 1 (DAPK1), and α‐synuclein levels.^16^, ^20^ ^–^ ^22^ Here, we designed a membrane‐permeable peptide (Tat‐SIRT5‐CTM) containing an 11‐amino acid segment encompassing the lysine 166 desuccinylation site of ANXA1 and the five upstream and downstream amino acids. We hypothesized that the synthetic peptide Tat‐SIRT5‐CTM can promote SIRT5 degradation via a lysosome‐mediated pathway, thus protecting neurons from ischemic brain injury.

Currently, we explored this hypothesis in preclinical experiments using an in vitro oxygen–glucose deprivation and reperfusion (OGD/R) cell model and an in vivo animal model of middle cerebral artery occlusion (MCAO). We first studied the effectiveness of Tat‐SIRT5‐CTM in inducing SIRT5 degradation. Next, we found that treatment with Tat‐SIRT5‐CTM inhibited the SIRT5‐ANXA1 interaction and blocked the desuccinylation of ANXA1 following OGD/R. Importantly, Tat‐SIRT5‐CTM suppressed nuclear transport of ANXA1 and increased its membrane accumulation and secretion, followed by inhibiting microglia‐induced neuroinflammation, ultimately alleviating neuronal damage in vitro. Moreover, administration of Tat‐SIRT5‐CTM rescued neuronal loss, decreased the infarct area, and improved sensorimotor and cognitive functions after ischemic stroke in vivo. We show proof‐of‐principle evidences for the use of this Tat‐SIRT5‐CTM peptide as a potentially effective new candidate for the therapy of ischemic stroke.

## 2 RESULTS

### 2.1 The Tat‐SIRT5‐CTM peptide leads to the lysosomal degradation of SIRT5

**FIGURE 1:**
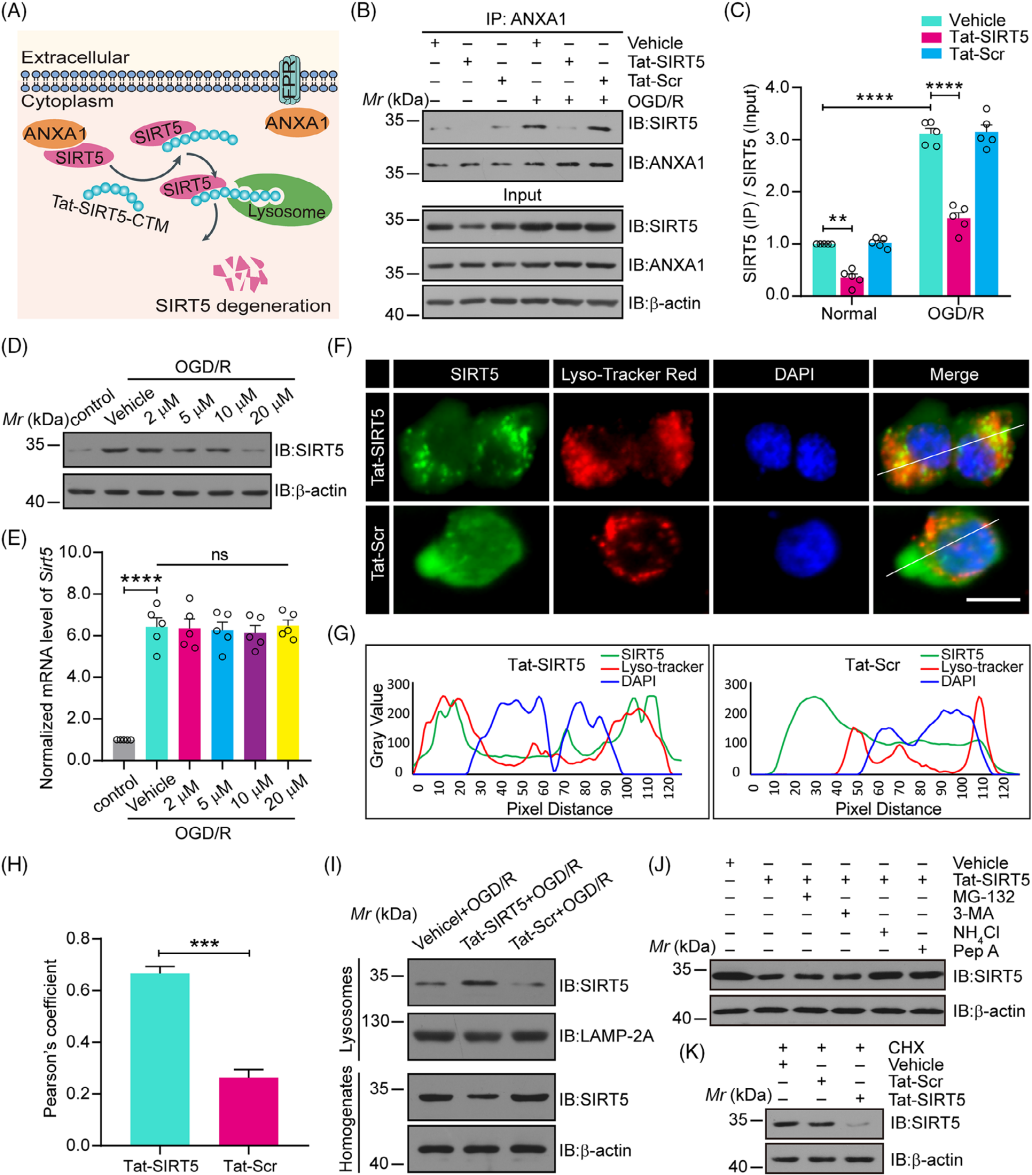
The Tat‐SIRT5‐CTM peptide inhibited the SIRT5‐ANXA1 interaction and dose‐dependently promoted SIRT5 degradation through the lysosomal pathway. (A) A schematic diagram showing that Tat‐SIRT5‐CTM inhibits the interaction of SIRT5 with ANXA1 and results in SIRT5 degradation. (B, C) Representative coimmunoprecipitation (co‐IP) and immunoblot results revealing the SIRT5‐ANXA1 interaction in primary cultured microglia (B) and quantitative analysis of the results (C). (D) Representative immunoblot showing the expression of SIRT5 in primary microglia treated with the indicated dose of Tat‐SIRT5‐CTM. (E) qRT‐PCR shows the mRNA levels of *Sirt5* in primary microglia. (F) The colocalization of SIRT5 with LysoTracker Red in primary microglia was analyzed by fluorescence microscopy. Scale bar = 10 μm. (G) The line of the panels shows the merged profiles of the fluorescent signal of SIRT5, LysoTracker Red, and DAPI signals along the white oblique line in the image. (H) Colocalization was quantitatively analyzed by Pearson's correlation analysis using ImageJ. (I) Representative immunoblot showing the level of SIRT5 in lysosomes. (J) Representative immunoblots shows effects of MG‐132, 3‐MA, NH_4_Cl, and Pep A on Tat‐SIRT5‐CTM mediated destabilization of SIRT5. (K) Representative immunoblots show effects of Tat‐SIRT5‐CTM‐mediated destabilization of SIRT5 on CHX treated microglia. The data are expressed as the mean ± SD and were obtained from at least three independent experiments. *M_r_*, relative molecular mass. ns: no significance; ***P < 0.001 and ****P < 0.0001.

Our previous study showed that SIRT5 bound to and desuccinylated ANXA1 at K166, which was sufficient to induce neuroinflammatory and neurological function damage after ischemic stroke. Moreover, SIRT5 knockdown via shRNA protects against ischemic brain injury.^15^ To design a peptide for SIRT5 knockdown as a potential clinically applicable treatment for ischemic stroke, we synthesized a short peptide (Tat‐SIRT5‐CTM) composed of an 11‐amino acid sequence encompassing the K166 desuccinylation site of ANXA1 and the five upstream and downstream amino acids (amino acids 161 to 171; KRDLAKDITSD), which specifically binds to and then leads to the lysosomal degradation of SIRT5 (Figure 1A). Our goal was to test the hypothesis that Tat‐SIRT5‐CTM is effective in protecting against ischemic stroke.

First, we confirmed the cellular source and localization of SIRT5 and ANXA1 under physiological conditions and after MCAO. Consistent with our previous study,^15^ double immunofluorescence staining of brain sections revealed that SIRT5 and ANXA1 were predominantly expressed in NeuN‐positive neurons and Iba1‐positive microglia, and an increase in SIRT5 and ANXA1 expression was observed in MCAO mice. However, there was rather poor colocalization of SIRT5 or ANXA1 with the astrocytic marker GFAP (Figures S1 and S2). These data suggested that SIRT5 and ANXA1 were primarily expressed in microglia and neurons. Furthermore, as shown in Figure S2, ANXA1 is primarily located in the cytoplasm of microglia and neurons under normal conditions, whereas the distribution of ANXA1 in the nucleus is markedly increased after MCAO. These results are consistent with our previous studies.^15^, ^23^, ^24^, ^25^, ^26^, ^27^, ^28^ We then examined the effect of Tat‐SIRT5‐CTM on the binding of SIRT5 to ANXA1, and a Tat‐Scramble‐CTM (Tat‐Scr‐CTM) peptide was used as a negative control. As expected, Tat‐SIRT5‐CTM significantly interfered with the interaction between SIRT5 and ANXA1 (Figure 1B, C). Additionally, increasing the concentration of Tat‐SIRT5‐CTM from 2 to 20 μM led to dose‐dependent SIRT5 degradation (Figure 1D), while it had little impact on the mRNA level of *Sirt5* in microglia (Figure 1E). Since SIRT5 is also expressed in neurons, we then explored the effect of Tat‐SIRT5‐CTM on the SIRT5 expression in primary cultured neurons. As shown in Figure S3, Tat‐SIRT5‐CTM treatment significantly decreased the protein level of SIRT5, but had little effect on its mRNA level in neurons after OGD/R. Furthermore, immunofluorescence staining showed that treating microglia with Tat‐SIRT5‐CTM increased the accumulation of SIRT5 in lysosomes (Figure 1F–H). The western blotting results also confirmed that Tat‐SIRT5‐CTM treatment indeed increased the level of SIRT5 in lysosomes after OGD/R (Figure 1I). Moreover, Tat‐SIRT5‐CTM–mediated clearance of SIRT5 was completely abolished by application with ammonium chloride (NH_4_Cl), which inhibits lysosome degradation,^29^ and pepstatin A (Pep A), which inhibits the two primary lysosomal proteases cathepsins D and E,^21^ but not the proteasome inhibitor MG‐132 or the macroautophagy inhibitor 3‐methyladenine (3‐MA) (Figure 1J). Tat‐SIRT5‐CTM also promoted SIRT5 degradation in primary microglia after treatment with cycloheximide (CHX), a protein synthesis inhibitor (Figure 1K). Collectively, these data demonstrated that Tat‐SIRT5‐CTM regulates SIRT5 degradation via chaperone‐mediated autophagy and the lysosomal degradation pathway but not the ubiquitin‒proteasome system.

To clarify the specificity of the effect of Tat‐SIRT5‐CTM on SIRT5 degradation, we explored the effects of Tat‐SIRT5‐CTM on the expression of other members of the SIRT family. As presented in Figure S4, Tat‐SIRT5‐CTM reduced the protein level of SIRT5 but had no effects on the protein level of other members of the SIRT family. All these results suggested that Tat‐SIRT5‐CTM effectively inhibits the binding of SIRT5 to ANXA1 and specifically induces the lysosomal degradation of SIRT5.

### 2.2 Tat‐SIRT5‐CTM blocks the desuccinylation of ANXA1, increasing its membrane accumulation and inducing ANXA1‐FPR2 binding

**FIGURE 2:**
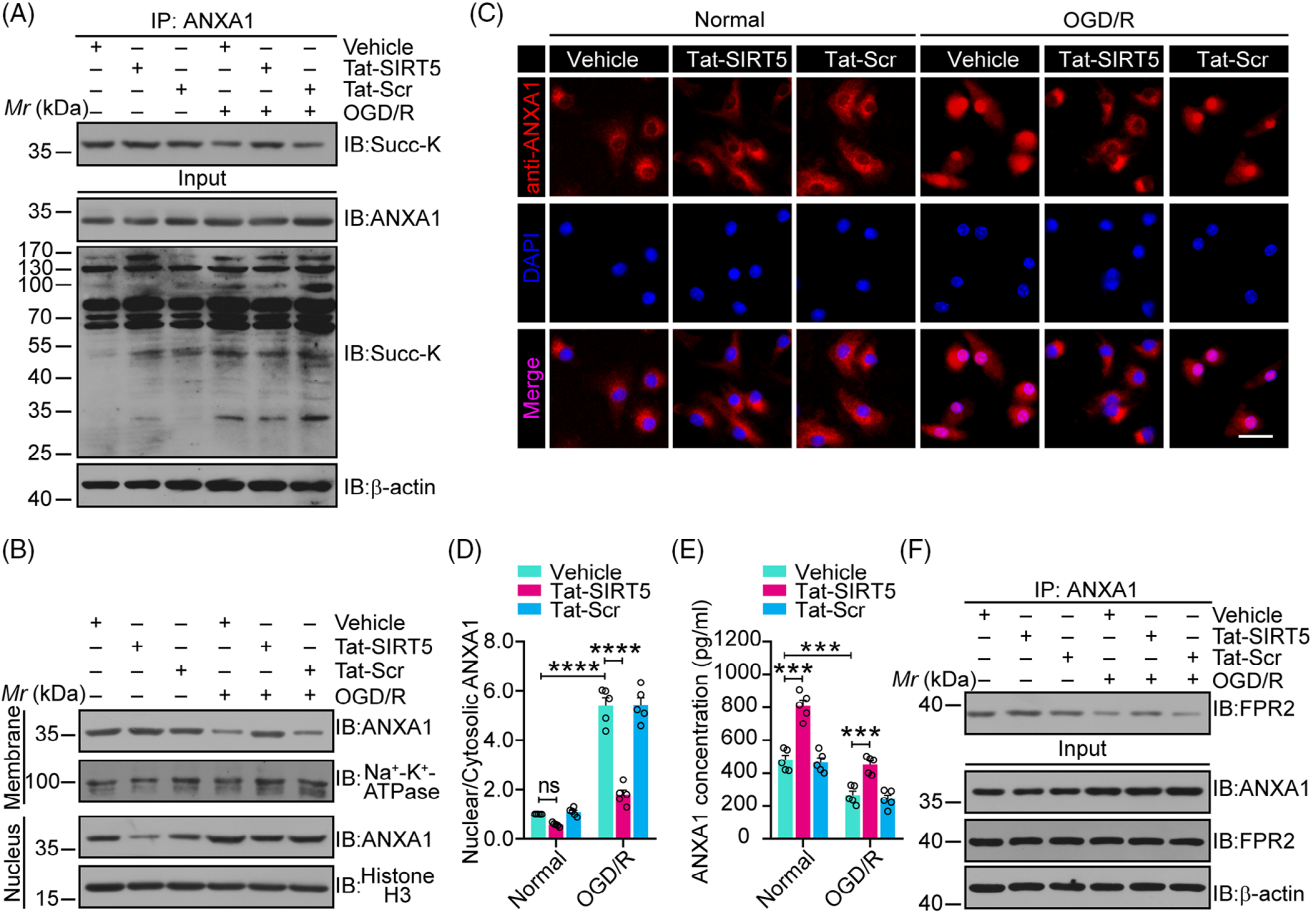
Tat‐SIRT5‐CTM inhibited ANXA1 nuclear localization but increased its membrane translocation and secretion during OGD/R. (A) Representative co‐IP assay results revealing ANXA1 succinylation level. (B) Representative immunoblot demonstrating the expression of ANXA1 in different subcellular compartments. Na^+^‐K^+^‐ATPase and histone H3 were used as membrane and nucleus loading controls, respectively. (C, D) Representative images of immunofluorescence staining revealing the subcellular localization of ANXA1 (C) and statistical analysis of nuclear/cytoplasmic ANXA1 expression (D). Scale bar = 20 μm. (E) ELISA results demonstrating the level of ANXA1 secretion. (F) Representative co‐IP results revealing the degree to which ANXA1 and FPR2 interacted. The data are expressed as the mean ± SD and were obtained from at least three independent experiments. ***P < 0.001 and ****P < 0.0001.

We have previously shown that SIRT5 desuccinylates ANXA1 to induce an increase in its nuclear transport and a decrease in its membrane recruitment, leading to decreased ANXA1 secretion.^15^ We then investigated whether Tat‐SIRT5‐CTM increases ANXA1 succinylation levels. Primary microglial cells were exposed to OGD/R and then treated with Tat‐SIRT5‐CTM or Tat‐Scr‐CTM at a concentration of 20 μM. As expected, compared with Tat‐Scr‐CTM, Tat‐SIRT5‐CTM obviously increased ANXA1 succinylation during OGD/R (Figure 2A). We also examined the cellular distribution of ANXA1 in microglia, and the results revealed that ANXA1 was mainly restricted to the nucleus after OGD/R. After Tat‐SIRT5‐CTM treatment, ANXA1 was upregulated in the membrane but not in the nucleus (Figure 2B). In addition, immunofluorescence assays showed that Tat‐SIRT5‐CTM inhibited OGD/R‐induced nuclear transport of ANXA1 in microglia (Figure 2C, D). Furthermore, we measured the secretion of ANXA1 into the supernatant of microglial cell cultures. Enzyme‐linked immunosorbent assay (ELISA) demonstrated that the secretion of ANXA1 was obviously upregulated following Tat‐SIRT5‐CTM treatment (Figure 2E). Importantly, Tat‐SIRT5‐CTM greatly increased the interaction of ANXA1 with formyl peptide receptor type 2 (FPR2) (Figure 2F). Collectively, these results indicated that Tat‐SIRT5‐CTM functions as a positive regulator to inhibit ANXA1 desuccinylation, decreases its nuclear translocation, and increases its membrane accumulation, leading to increased ANXA1 secretion.

### 2.3 Tat‐SIRT5‐CTM inhibits the expression of inflammatory cytokines in microglia after OGD/R

**FIGURE 3:**
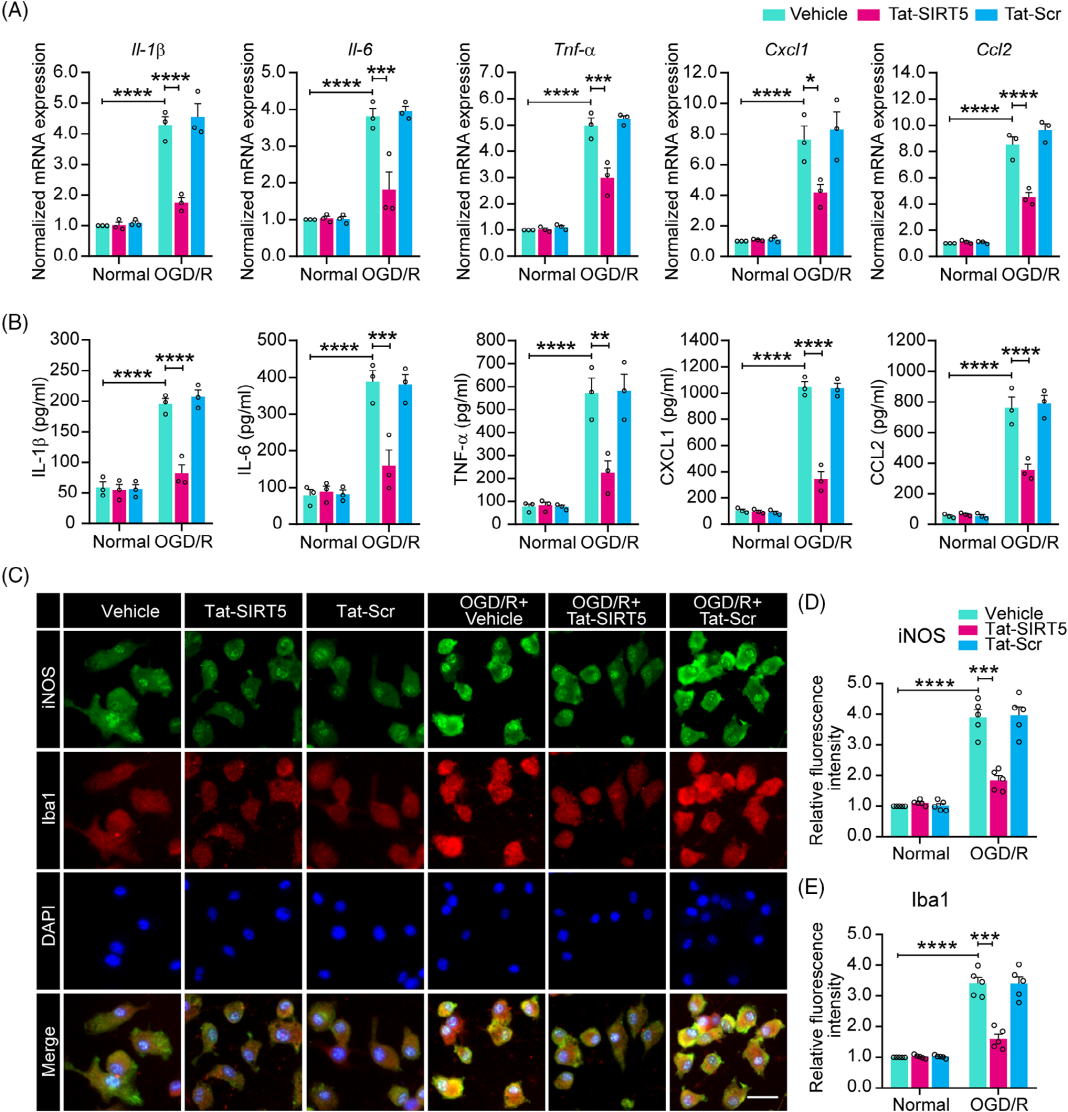
Tat‐SIRT5‐CTM inhibits OGD/R‐induced production of proinflammatory mediators in microglia. (A) qRT‒PCR results demonstrating the mRNA levels of *Il‐1β, Il‐6*, *Tnf‐α, Cxcl1*, and *Ccl2* in primary cultured microglia under normal and OGD/R conditions. (B) ELISA results revealing the protein expression levels of IL‐1β, IL‐6, TNF‐α, Cxcl1, and Ccl2 in the supernatants of microglia under normal and OGD/R conditions. (C) Immunofluorescence staining of iNOS, Iba‐1 with DAPI under normal and OGD/R conditions. Scale bars = 20 μm. (D–E) The fluorescence intensity of iNOS (D) and Iba‐1 (E). The data are expressed as the mean ± SD and were obtained from three or five independent experiments. *P < 0.05, **P < 0.01, ***P < 0.001, and ****P < 0.0001.

A previous study suggested that SIRT5 increases the OGD/R‐induced production of inflammatory cytokines.^15^ Thus, we tested the hypothesis that Tat‐SIRT5‐CTM inhibits microglia‐induced neuroinflammation. First, quantitative real‐time polymerase chain reaction (qRT‒PCR) revealed that Tat‐SIRT5‐CTM greatly decreased the mRNA expression of proinflammatory mediators, including interleukin‐1β (IL‐1β), interleukin‐6 (IL‐6), tumor necrosis factor‐α (TNF‐α), C‐X‐C motif chemokine ligand 1 (CXCL1), and C‐C motif chemokine ligand 2 (CCL2), under OGD/R conditions (Figure 3A). Similarly, the ELISA revealed that the release of the cytokines and chemokines corresponded to their mRNA expression (Figure 3B). In addition, western blotting confirmed that the expression of the proinflammatory mediators inducible nitric oxide synthase (iNOS) and CD16/32 in microglia were substantially decreased after Tat‐SIRT5‐CTM treatment following OGD/R (Figure S5). Correspondingly, immunofluorescence staining for iNOS and Iba‐1 was conducted in microglia. The results showed that Tat‐SIRT5‐CTM significantly decreased the fluorescence intensity of iNOS and Iba‐1 after OGD/R (Figure 3C–E). Taken together, these findings demonstrated that Tat‐SIRT5‐CTM suppresses proinflammatory mediators expression in microglia after OGD/R.

### 2.4 Tat‐SIRT5‐CTM alleviates OGD/R‐induced neuronal damage

**FIGURE 4:**
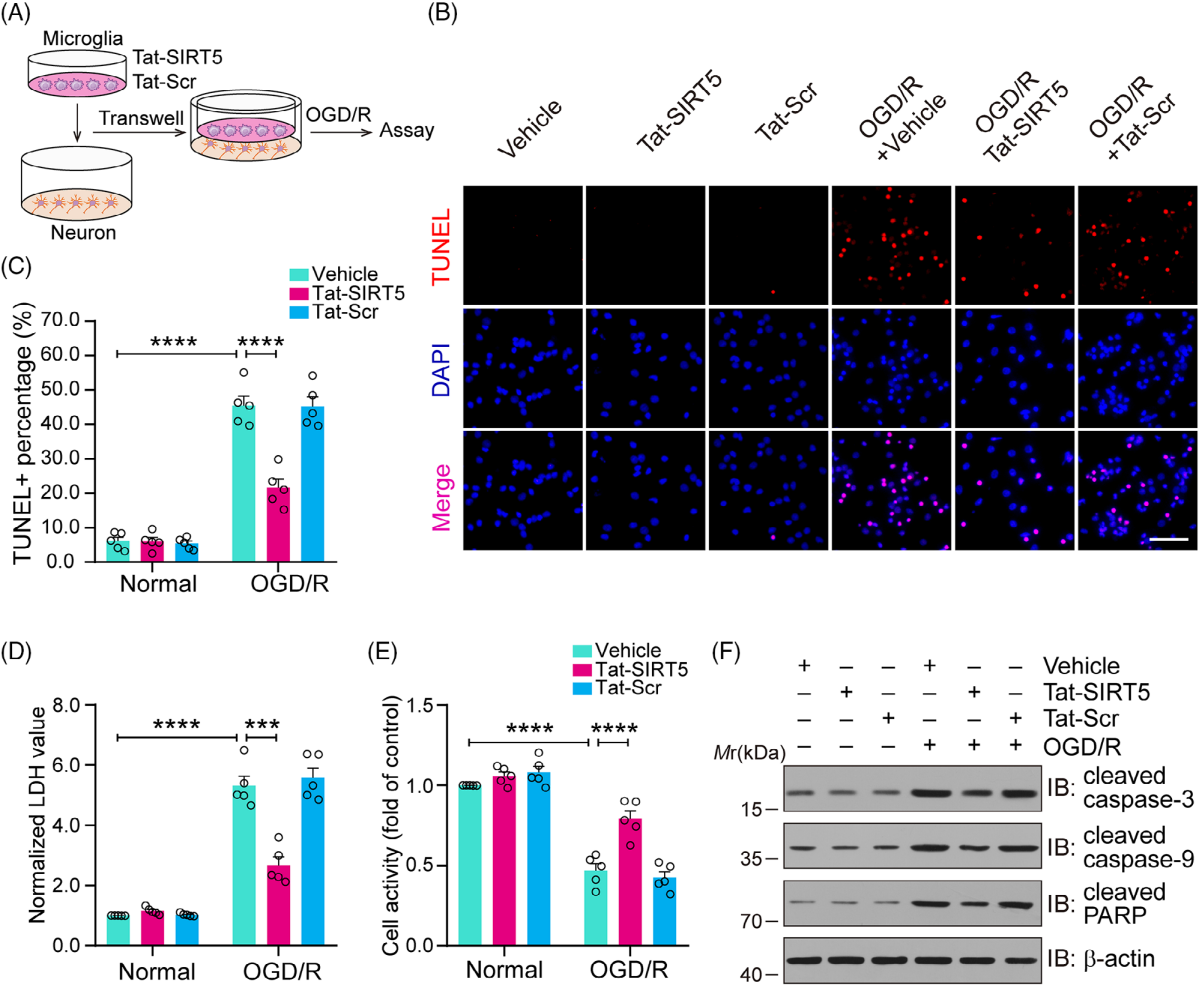
Application of microglia with Tat‐SIRT5‐CTM alleviates OGD/R‐induced neuronal death. (A) Primary microglia were treated with Tat‐SIRT5‐CTM or Tat‐Scr‐CTM peptide and then cocultured with primary neurons in a Transwell system. Schematic representation as shown above. (B, C) Representative images of TUNEL staining and (B) quantitative analysis of TUNEL‐positive cells (C). (D) Results of the LDH release assay showing the degree of neuronal damage. (E) Results of the CCK‐8 assay showing neuronal viability. (F) Representative immunoblot representing the levels of proapoptotic molecules in neurons. The data are expressed as the mean ± SD and were obtained from five independent experiments. *P < 0.05, **P < 0.01, ***P < 0.001, and ****P < 0.0001.

Next, as shown in Figure 4A, we used a microglia‐neuron coculture system to further determine whether Tat‐SIRT5‐CTM can directly protect against neuronal damage induced by OGD/R through microglial regulation. First, TdT‐mediated dUTP‐X nick end labeling (TUNEL) staining was performed to assess neuronal death, and it was shown that Tat‐SIRT5‐CTM significantly decreased the percentage of TUNEL‐positive neurons (Figure 4B, C). Consistently, Tat‐SIRT5‐CTM significantly decreased lactate dehydrogenase (LDH) release (Figure 4D). Cell‐counting‐kit‐8 (CCK8) assays were performed to measure neuronal viability, and the results revealed that Tat‐SIRT5‐CTM promoted neuronal viability (Figure 4E). Finally, we conducted immunoblot assays to examine the protein level of proapoptotic molecules in neurons. The results demonstrated that Tat‐SIRT5‐CTM obviously suppressed the expression of these proteins under OGD/R conditions (Figure 4F). In conclusion, these results revealed that microglia treated with Tat‐SIRT5‐CTM can protect against neuronal damage under OGD/R conditions.

### 2.5 Tat‐SIRT5‐CTM interrupts the SIRT5‐ANXA1 interaction and suppresses the inflammatory response in vivo

**FIGURE 5:**
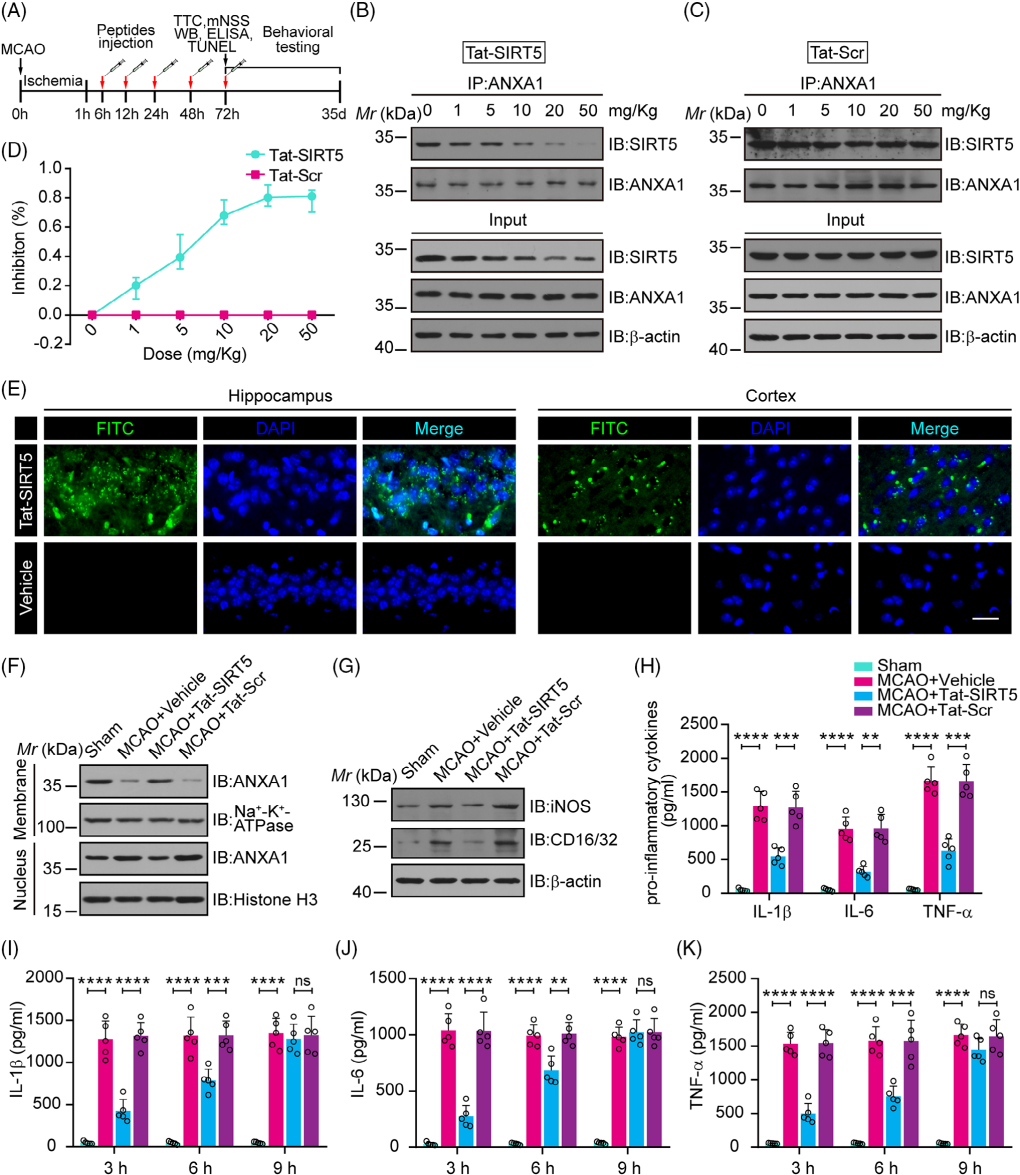
The Tat‐SIRT5‐CTM peptide blocks the SIRT5‐ANXA1 interaction and suppresses the inflammatory response in mice. (A) A diagram of the experimental timeline. (B, C) Representative blots from the co‐IP assays showing the dose‐dependent effects of Tat‐SIRT5‐CTM and Tat‐Scr‐CTM on the SIRT5‐ANXA1 binding. (D) Dose‒response curves of the inhibitory effects of the peptides on the SIRT5‐ANXA1 interaction. (E) Penetration of FITC‐Tat‐SIRT5‐CTM into hippocampal and cortical tissues. FITC was detected in nonfixed tissue slices under fluorescence microscopy. Scale bars, 50 μm. (F) Representative immunoblot revealing the expression of ANXA1 in the membrane and nuclear fractions of mouse peri‐infarct tissues. (G) Representative immunoblot showing the expression of iNOS and CD16/32 in mouse peri‐infarct tissues. (H–K) ELISA results showing the expression of IL‐1β, IL‐6, and TNF‐α in mouse peri‐infarct tissues. The data are expressed as the mean ± SD. *n* = 5 mice per group. *P < 0.05, **P < 0.01, ***P < 0.001, and ****P < 0.0001.

We then investigated whether Tat‐SIRT5‐CTM induces ischemic tolerance in vivo. A transient focal ischemic stroke animal model was established, and a series of histological and behavioral assessments were performed at various time points after intravenous injections of Tat‐SIRT5‐CTM (Figure 5A). First, different doses (1, 5, 10, 20, or 50 mg/kg) of the indicated peptides were injected into the animals immediately after the onset of MCAO. Coimmunoprecipitation (co‐IP) showed that Tat‐SIRT5‐CTM at a dose of 20 mg/kg efficiently decreased the binding of SIRT5 with ANXA1. In addition, in the input lanes of co‐IP assays, we repeatedly detected that Tat‐SIRT5‐CTM led to SIRT5 degradation in a dose‐dependent manner, while Tat‐Scr‐CTM had little effect on SIRT5 (Figure 5B–D). Consequently, a dose of 20 mg/kg was adopted in the remainder of the research. Then, immunofluorescence staining showed that fluorescein isothiocyanate (FITC)‐labeled Tat‐SIRT5‐CTM was effectively distributed in hippocampal and cortical tissues (Figure 5E). Next, we explored the subcellular localization of ANXA1. The results revealed that Tat‐SIRT5‐CTM greatly upregulated the level of ANXA1 in the membrane fraction but increased the ANXA1 level to a lesser extent in the nucleus (Figure 5F, Figure S6A). Furthermore, immunoblot assays revealed that Tat‐SIRT5‐CTM markedly inhibited the expression of iNOS and CD16/32 (Figure 5G, Figure S6B, C). In addition, ELISA showed that Tat‐SIRT5‐CTM significantly decreased the production of IL‐1β, IL‐6, and TNF‐α (Figure 5H). All the above data demonstrated that Tat‐SIRT5‐CTM inhibits the ANXA1‐SIRT5 interaction, increases ANXA1 secretion, and ultimately inhibits the inflammatory response in vivo.

Our experiments suggest that administration of Tat‐SIRT5‐CTM immediately after the onset of MCAO can exert neuroprotection. However, only a small proportion of patients can be treated immediately after stroke. We therefore evaluated whether the use of Tat‐SIRT5‐CTM has wider applicability. To explore the effective time window for intervention, we treated the Tat‐SIRT5‐CTM peptide (20 mg/kg, i.v.) to mice at three time points (3, 6, and 9 h after reperfusion). As shown in Figure 5I–K, when administered 3‐ or 6‐h following reperfusion, Tat‐SIRT5‐CTM was effective in inhibiting the production of inflammatory cytokines. However, no change was observed in the mice that received Tat‐SIRT5‐CTM 9 h after reperfusion. Collectively, these data suggested that 20 mg/kg may be an effective dose of Tat‐SIRT5‐CTM and that 6 h after reperfusion is the therapeutic time window for this peptide.

### 2.6 Tat‐SIRT5‐CTM alleviates ischemia‐induced neuronal damage, reduces the infarct size, and improves neurological outcomes after ischemic stroke

**FIGURE 6:**
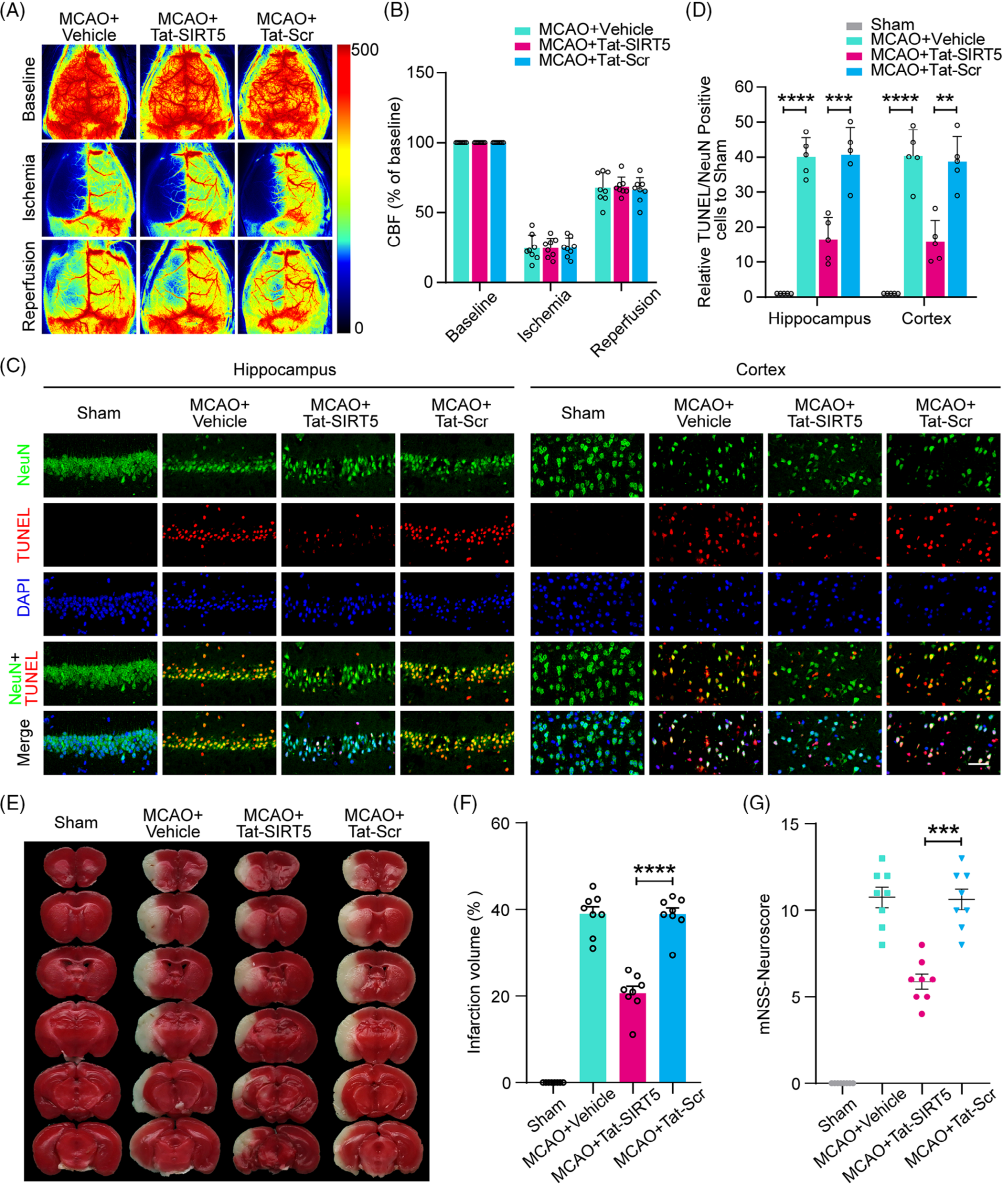
The Tat‐SIRT5‐CTM peptide alleviated ischemic brain injury in vivo. (A) CBF assessed by two‐dimensional laser speckle imaging before and during MCAO and reperfusion. (B) The results are presented as the percent change from baseline. (C) Representative images revealing TUNEL‐positive cells in the mouse peri‐infarct hippocampus and cortex. (D) Statistical analysis was performed by counting the number of NeuN^+^ and TUNEL^+^ cells. (E) Representative images of TTC‐stained tissues from mice treated with the Tat‐SIRT5‐CTM or Tat‐Scr‐CTM peptide. (F) Statistical analysis of the infarct volume ratio. (G) mNSS showing the extent of neurological deficits at 72 h after reperfusion. The data are expressed as the mean ± SD. *n* = 5 or 8 mice for each group. **P < 0.01, ***P < 0.001, and ****P < 0.0001.

To investigate the neuroprotective effects of Tat‐SIRT5‐CTM in mice subjected to focal ischemic stroke injury, we first monitored cerebral blood flow (CBF) via a laser speckle imaging system. During MCAO surgery and reperfusion, the mice administered Tat‐SIRT5‐CTM and those administered Tat‐Scr‐CTM displayed equivalent regional CBF (Figure 6A, B). Then, 72 h following ischemic insult and peptide injection, TUNEL assay was applied to evaluate neuronal apoptosis, and the results showed that 1 h of MCAO followed by 72 h of reperfusion resulted in a significant decrease in the number of surviving cells and an increase in the number of apoptotic cells in the mouse peri‐infarct hippocampus and cortex, while Tat‐SIRT5‐CTM exerted neuroprotective effects (Figure 6C, D). Moreover, we stained the infarct tissues with 2,3,5‐triphenyltetrazolium chloride (TTC) to detect the infarct volume. The results demonstrated that Tat‐SIRT5‐CTM successfully reduced the cerebral infarct size (Figure 6E, F). In addition, we employed the modified neurological severity scores (mNSS) to measure neurological impairment. The mice application with Tat‐SIRT5‐CTM displayed a better neurological score (Figure 6G). These results suggested that Tat‐SIRT5‐CTM significantly decreases neuronal damage, the infarct size, and neurological deficits after ischemic brain injury.

### 2.7 Tat‐SIRT5‐CTM preserves long‐term neurobehavioral functions after ischemic brain injury

**FIGURE 7:**
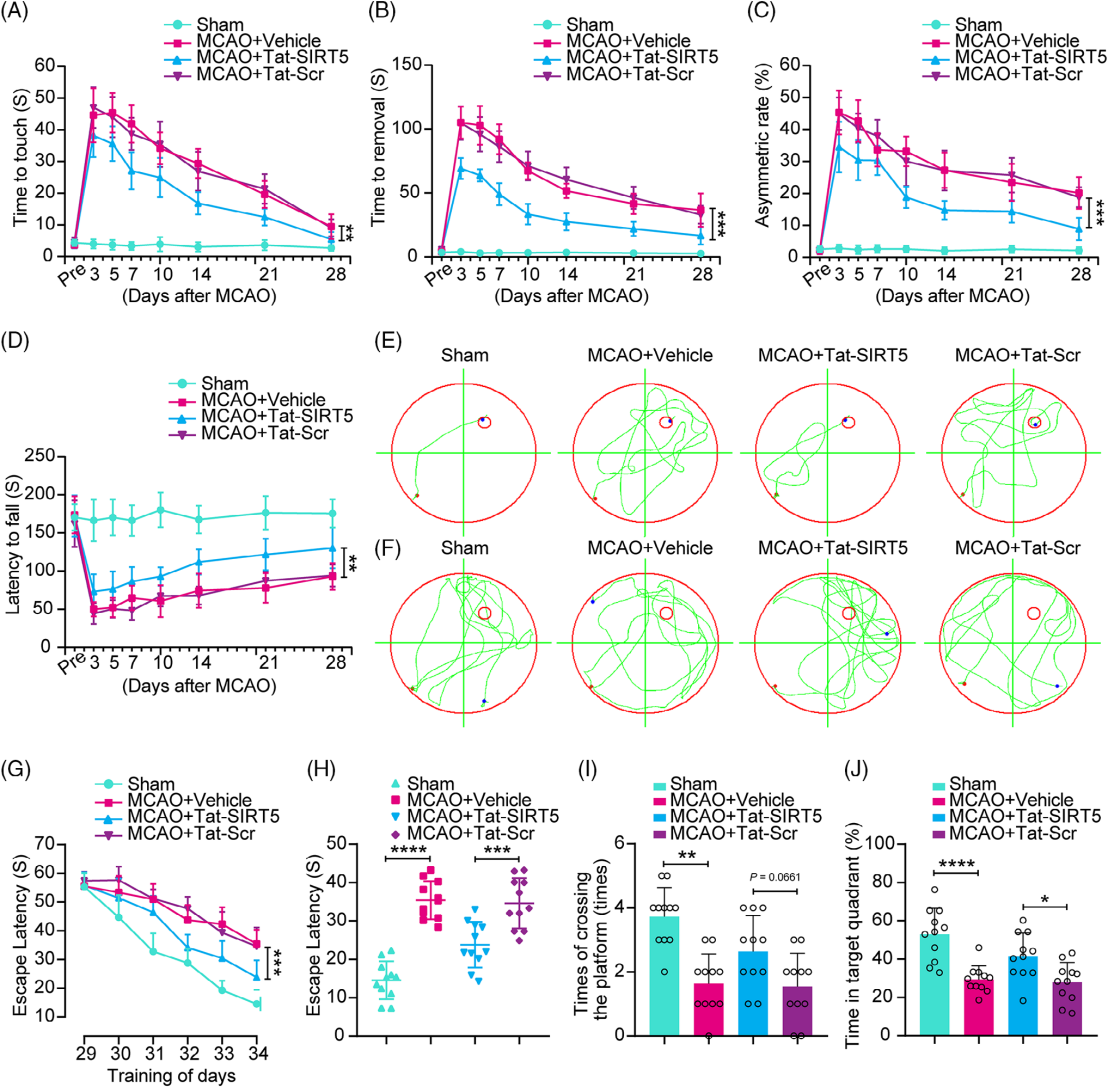
Administration of Tat‐SIRT5‐CTM improves long‐term sensorimotor and cognitive functions in mice. (A–D) Sensorimotor deficits were determined by a series of behavioral tests. (A, B) Adhesive test. (C) Cylinder test. (D) Rotarod test. (E–J) Results from the latency trial (G and H) and probe trial (I and J) of the MWM test. (E, F) Representative swimming traces from the latency trials (E) and probe trials (F) of the MWM test. (G) The latency to reach a submerged platform on days 1−6 during the MWM test. (H) The time spent searching for the submerged platform on day 6 during the MWM test. (I) The total number of platform region crossings in the probe trial on day 7 during the MWM test. (J) Statistical analysis showing the percentage of time spent in the target quadrant in the probe trial on day 7. Quantitative analysis in panel (I) was assessed by the Kruskal–Wallis nonparametric test. Data in panel (H, J) were assessed by one‐way ANOVA followed by Tukey's post‐hoc test, and all others were determined by the RM ANOVA followed by Tukey's post hoc test. The data are expressed as the mean ± SD. *n* = 11 mice per group. *P < 0.05, **P < 0.01, ***P < 0.001, and ****P < 0.0001.

To assess sensorimotor functions following ischemic stroke, we first performed a battery of behavioral tests, including the adhesive test and the cylinder test. As shown in Figure 7A–C, MCAO resulted in severe impairment of sensorimotor functions in the vehicle group, as evidenced by an increase in the time to touch and remove the adhesive tape from the forepaws, as well as the increase in the asymmetry rate in the cylinder test, whereas Tat‐SIRT5‐CTM treatment facilitated neurological recovery after stroke. Next, we performed the rotarod test to assess motor function and found that the Tat‐SIRT5‐CTM‐treated mice took longer to fall off the rod (Figure 7D). Additionally, we tested the spatial learning and memory ability of mice using the Morris water maze (MWM) test. Representative traces from the latency trials and the probe trials are presented in Figure 7E and F. Compared to the sham controls, the MCAO animals took a much longer time to find the submerged platform and spent less time in the target quadrant. However, the Tat‐SIRT5‐CTM‐treated mice displayed substantial improvement of cognitive function (Figure 7G, H). On Day 7 of the probe trials, the Tat‐SIRT5‐CTM peptide‐treated animals crossed the platform more times and spent much more time in the target quadrant than Tat‐Scr‐treated mice (Figure 7I, J). In summary, these results demonstrated that Tat‐SIRT5‐CTM improves long‐term neurological function after cerebral ischemia.

To further develop Tat‐SIRT5‐CTM as a therapeutic candidate for cerebral ischemia, we then explored the safety profile of the peptide. Tat‐SIRT5‐CTM was injected into nonischemic mice at a dosage of 20 or 100 mg/kg/day for seven consecutive days. We first investigated the effect of Tat‐SIRT5‐CTM on neuronal death and cognitive function in mice. As presented in Figures S7 and S8, Tat‐SIRT5‐CTM had little impact on neuronal death or neurobehavioral performance. Furthermore, the levels of liver transaminase and renal dysfunction biomarkers were not elevated (Figure S9). Collectively, these data indicated that Tat‐SIRT5‐CTM caused little toxicity in mice.

## 3 DISCUSSION

In this research, we have provided compelling evidence that Tat‐SIRT5‐CTM may have therapeutic promise for the clinical treatment of ischemic stroke because it rapidly decreases the protein level of SIRT5. We first showed that Tat‐SIRT5‐CTM can interrupt the SIRT5‐ANXA1 interaction, trigger the lysosomal degradation of SIRT5, suppress the nuclear transport of ANXA1, and increases its membrane accumulation and release. We also provided evidence that peptide‐induced SIRT5 knockdown is relevant to the protection of neurons from microglia‐induced neuroinflammation in an in vitro culture model of cerebral ischemia. Furthermore, using an animal model of focal brain ischemia, we were able to show the therapeutic potential of systemic administration of Tat‐SIRT5‐CTM as a treatment for ischemic stroke. Our findings not only establish SIRT5 as a therapeutic target for ischemic stroke but also show that the Tat‐SIRT5‐CTM peptide designed in the present study may represent a potential therapeutic candidate for ischemic stroke.

Cell‐penetrating peptides have been employed in basic preclinical studies and clinical trials.^30^ Tat is a cell‐penetrating peptide that can rapidly and efficiently transport a wide variety of proteins, peptides, and small medicines across the BBB and plasma membrane.^31^, ^32^ A wide variety of Tat conjugated peptides have shown promising effects in a variety of disease models and, in some cases, have been investigated in clinical studies.^33^, ^34^, ^35^, ^36^, ^37^, ^38^ It was recently proven through a phase 3 clinical trial that a Tat‐fused peptide is not only safe but also effective in preventing ischemic damage to human neurons.^39^ Nerinetide, also known as Tat‐NR2B9c or NA‐1, comprises the last nine amino acids of the carboxyl tail of GluN2B. By specifically binding to PSD95, this peptide protects neurons from NMDAR‐mediated excitotoxicity. In cynomolgus macaques and humans, Nerinetide has been demonstrated to effectively and safely relieve cerebral ischemic injury.^40^, ^41^, ^42^, ^43^ We hope that this Tat‐SIRT5‐CTM peptide can also be successfully translated to the clinic as a new and effective treatment for ischemic stroke in human patients. In this research, we showed that injection of Tat‐SIRT5‐CTM led to effective transport of the peptide into brain tissue and that administration of the peptide within 6 h of stroke produced a therapeutic effect; this is in contrast to the therapeutic time window of tPA, which is < 4.5 h after stroke onset.^44^ Nonetheless, more detailed studies are needed to determine the exact therapeutic window of Tat‐SIRT5‐CTM. As Tat‐SIRT5‐CTM exerts neuroprotective effects against ischemia when injected after reperfusion, it is also important to assess its effect when administered prior to reperfusion.

Our SIRT5 knockdown peptide (Tat‐SIRT5‐CTM) provides several advantages over existing methods such as genetic manipulation or small molecule inhibitors. Frist, although shRNA‐mediated knockdown of SIRT5 has also been demonstrated to be effective in ischemic stroke models,^15^ its clinical translations are constrained by its inability to pass through the BBB. Delivery of shRNAs into the brain is primarily accomplished by invasive intracerebroventricular injection or viral infection, which may not be practical for the treatment of human patients. However, our peptide‐based approach delivers cargo into brain following noninvasive systemic administration using a Tat‐mediated protein transduction mechanism, making it much simpler and more effectively. The high potency of the peptide in knocking down SIRT5 in the brain and its potent neuroprotective efficacy in animal models of cerebral ischemia clearly demonstrate its effectiveness. Second, Tat‐SIRT5‐CTM‐mediated knockdown has a clear temporal advantage over genetic manipulation. By hijacking the endogenous lysosomal degradation system in the cell, the peptide‐based method can produce a rapid and robust degradation of target protein within a few hours.^16^ Third, since the interactions between peptide and target protein is extremely specific, the Tat‐SIRT5‐CTM peptide was specific for its own intended target protein SIRT5, with no off‐target effects, as Tat‐SIRT5‐CTM had no effects on the protein levels of the other members of SIRT family. On the other hand, it should be noted that SIRT5 is also expressed in tissues and organs outside the brain. Thus, whether any of potential therapeutic effects of systemic application of Tat‐SIRT5‐CTM can result in a knockdown of SIRT5 in any of the peripheral tissues merits further study. Furthermore, since SIRT5 is an important desuccinylase in the cell, approach interfering with its activity or promoting its protein degradation may inevitably affect the succinylation level of other substrate proteins, and thus may have effects on the normal function of these substrates and downstream signaling pathways. Therefore, aside from impacts on neuroinflammation and neuroprotection, one unique consideration that the other cellular processes that affected by Tat‐SIRT5‐CTM will need to be investigated.

Previous research has indicated that ANXA1 has several biological functions depending on its subcellular distribution.^24^, ^25^, ^27^, ^45^, ^46^, ^47^ Membrane trafficking and release of ANXA1 have anti‐inflammatory effects and alleviate neuronal damage caused by OGD/R.^15^, ^27^ Nevertheless, evidences also suggest that the nuclear transport of ANXA1 may promote proinflammatory factor expression in microglia after ischemia.^45^, ^48^ The findings of this study suggest that Tat‐SIRT5‐CTM regulates the subcellular distribution of ANXA1. We found that Tat‐SIRT5‐CTM increases ANXA1 membrane trafficking and release and enhances the interaction of ANXA1 with FPR2. There is increasing evidence, showing that the subcellular distribution of ANXA1 is strictly regulated by posttranslational modifications (PTMs), including SUMOylation, succinylation, and phosphorylation.^23^, ^26^ The interaction of various PTMs, which impact protein function synergistically or antagonistically, has been widely investigated.^49^ Our previous study demonstrated that desuccinylation of ANXA1 by SIRT5 may increase the Sentrin/SUMO‐specific protease 6 (SENP6)‐ANXA1 interaction and reduce ANXA1 SUMOylation levels, thereby promoting the nuclear translocation of ANXA1.^15^ Protein kinase C (PKC)‐, epidermal growth factor receptor (EGFR)‐, and transient receptor potential melastatin 7 (TRPM7)‐mediated phosphorylation has also been demonstrated to play important roles in ANXA1 subcellular localization regulation.^23^, ^45^, ^50^ Therefore, whether Tat‐SIRT5‐CTM‐mediated ANXA1 membrane trafficking has effects on its SUMOylation or other PTMs needs further investigation.

Moreover, our previous study reported that the cell‐penetrating peptide Tat‐NTS inhibits the binding of ANXA1 to importin β, significantly inhibiting the nuclear transport of ANXA1 in neurons and thereby protecting against ischemic stroke‐induced neuronal damage.^51^, ^52^ However, the present study revealed that Tat‐SIRT5‐CTM targets the SIRT5‐ANXA1 complex, resulting in SIRT5 degradation via the lysosomal pathway, which eventually alleviates microglia‐induced neuroinflammation and neuronal injury following ischemic stroke.^20^ As the Tat‐NTS and Tat‐SIRT5‐CTM peptides act through different mechanisms in different cells, we supposed that a combination of the two different peptides would have greater efficacy. Of course, more detailed studies remain to be validated the effects of this combination treatment. In addition, we mainly focused on the effects of Tat‐SIRT5‐CTM on microglia‐induced neuroinflammation after ischemic stroke in this study. As SIRT5 is also expressed in neurons (Figure S1), and we found that Tat‐SIRT5‐CTM treatment can also significantly decrease the protein level of SIRT5 in primary cultured neurons after OGD/R (Figure S3). On the other hand, Tat peptides enter multiple cell types simultaneously in a nonselective manner;^53^ therefore, Tat‐SIRT5‐CTM can also be taken up by neurons in the brain when systemic administration via i.v. injection, making more detailed studies necessary to determine the directly effects of Tat‐SIRT5‐CTM treatment on neurons.

There are several limitations to this study. First, the pharmacokinetics parameters of Tat‐SIRT5‐CTM have not been investigated. Therefore, more pharmacokinetic and pharmacodynamic studies in relevant animal models are needed to clarify the optimal dosage, bioavailability, and half‐life of Tat‐SIRT5‐CTM. Second, because peptides require blood flow for delivery to the site of injury, we tested only an animal model of ischemia‒reperfusion model. There are no data yet on the effect of the peptide affect injury if reperfusion cannot be reestablished. More research works are required to clarify the protective impact of Tat‐SIRT5‐CTM on permanent occlusion models. Third, in contrast to stroke patients, the animals used as stroke models in our experiment were young and free of notable comorbidities. This may make it challenging to directly estimate the therapy efficacy the peptide in stroke patients. Finally, even though we investigated mixed‐sex primary neuronal cultures, the in vivo tests were exclusively carried out on males. Whether Tat‐SIRT5‐CTM has a sex‐dependent effect remains to be explored. Such constraints should be considered when designing clinical investigations.

In summary, our investigation confirmed that a small peptide, Tat‐SIRT5‐CTM, specifically inhibits neuroinflammation following ischemic stroke. We found that Tat‐SIRT5‐CTM increased ANXA1 membrane accumulation and release by disrupting the binding of SIRT5 to ANXA1 and promoting SIRT5 degradation via a lysosome‐mediated pathway, eventually leading to decreased production of proinflammatory mediators. Furthermore, in vitro and in vivo evidence revealed that Tat‐SIRT5‐CTM reduced neuronal damage and the infarct volume and improved long‐term neurological functional outcomes following ischemic stroke. In conclusion, the fusion peptide Tat‐SIRT5‐CTM may be a promising therapeutic approach for the clinical treatment of ischemic stroke and possibly other types of neuroinflammatory disorders.

## 4 MATERIALS AND METHODS

### 4.1 Animals

Male C57BL/6JNifdc mice aged 8 weeks and weighing 22−25 g were obtained from Charles River (Beijing Office, China). The mice were given unrestricted access to food and drink while being maintained at a constant temperature of 22°C and a 12‐h light/dark cycle. All animal experiments were approved by the Experimental Animal Care and Use Committee of Tongji Hospital, Tongji Medical College, and Huazhong University of Science and Technology. Experimental procedures, data analysis, documentation, and reporting adhere to the Animal Research: Reporting of In Vivo Experiments (ARRIVE) guidelines.^54^ Genuine efforts were made to minimize animal suffering and sacrifice. The animals were randomly divided into different groups using a random number generator.^55^ The experiments were conducted by researchers who were unaware of the group assignments. Power analysis and sample size (PASS) software was used to analyze preexperimental data with a significance level of = 0.05 and a power of 80% to identify significant differences. For animal experiments, the number of mice in each group was determined in accordance with the numbers reported in published papers or our previous experiments, and the number of mice in each group are given in the figure legends.

### 4.2 Reagents and antibodies

Anti‐ANXA1 (sc‐12740, 1:1000), anti‐FPR2 (sc‐100585, 1:1000), and anti‐β‐actin (sc‐47778, 1:1000) primary antibodies were purchased from Santa Cruz Biotechnology (Dallas, TX, USA); anti‐SIRT5 (15122‐1‐AP, 1:1000) and anti‐iNOS (18985‐1‐AP, 1:500) antibodies were obtained from Proteintech Group (Wuhan, China); an anti‐CD16/32 antibody (AF1460, 1:500) was bought from R&D Systems (Minneapolis, MN, USA); anti‐Na^+^‐K^+^/ATPase (#9339, 1:1000), anti‐Histone H3 (#4499, 1:1000), anti‐cleaved PARP (#5625, 1:1000), anti‐cleaved caspase‐3 (#9664, 1:1000), and anti‐cleaved caspase‐9 (#20750, 1:1000) antibodies were purchased from Cell Signaling Technology (Danvers, MA, USA); anti‐Iba1 (ab283319, 1:100) and anti‐LAMP2A (ab125068, 1:1000) antibodies were obtained from Abcam (Boston, MA, USA); and anti‐Succ‐K (PTM‐401, 1:1000) was obtained from PTMBIO (Hangzhou, China). Agarose beads containing proteins A and G were obtained from Beyotime Biotechnology (Shanghai, China). Roche provided the protease inhibitor cocktail as well as the In Situ Cell Death Detection Kit (Basel, Switzerland). All other generic reagents were obtained from commercial sources and used exactly as received.

### 4.3 Peptide treatment

The Tat‐SIRT5‐CTM (YGRKKRRQRRR‐KRDLAKDITSD‐KFERQKILDQRFFE) and Tat‐Scr‐CTM (YGRKKRRQRRR‐KKRDIDTLSAD‐KFERQKILDQRFFE) peptides (95% purity) were produced by GL Biochem, Ltd. (Shanghai, China). For in vitro studies, the peptides were dissolved in double‐distilled water and then diluted as needed using complete medium. Following OGD, primary microglia underwent treatment with Tat‐SIRT5‐CTM or Tat‐Scr‐CTM for 24 h at a concentration of 20 μM. For in vivo studies, the mice received a tail vein injection of Tat‐SIRT5‐CTM, Tat‐Scr‐CTM, or saline at a dosage of 20 mg/kg at 6, 12, and 24 h after stroke onset or at the indicated time after reperfusion. For optimal outcomes, another two doses of the peptides were administered on the second and third days.

### 4.4 Coculture of neurons and microglia

As previously reported, neurons and microglia were cocultured.^56^ Primary neurons and microglia were isolated and cultured in a Transwell system (Corning, Tewksbury, MA, USA). To allow cytokines to diffuse, we cultured primary microglia and neurons in two chambers separated by a 0.4‐μm semipermeable membrane. First, we cultured the neurons in the bottom chamber and the microglia in the top chamber. After microglia were treated with Tat‐SIRT5‐CTM for 2 h, the Transwell insert was placed in the chamber containing the neurons, and the supernatants were replaced with fresh medium. Afterward, the cocultured microglia and neurons were challenged with OGD/R.

### 4.5 OGD/R

OGD/R was performed as previously reported.^24^ For OGD, the cells were washed three times and then maintained in glucose‐free Dulbecco's modified Eagle medium (DMEM, Gibco, Gaithersburg, MD, USA) that had been prewarmed to 37°C, which was preequilibrated with medium that was oxygen‐depleted; and then moved to a three‐gas hypoxic incubator containing 1 % O_2_, 94 % N_2_, and 5 % CO_2_ at 37°C for 60 min. The culture medium was thereafter substituted with normal DMEM with glucose, and the cells were incubated for an additional 24 h at 37°C in 5% CO_2_ for reperfusion. The cells were then collected for analysis.

### 4.6 Transient focal cerebral ischemia

To induce focal ischemic stroke, mice were subjected to a left MCAO surgery for 60 min as described previously by our group.^48^ The experimental procedures were conducted in accordance with the Ischaemia Models: Procedural Refinements Of in Vivo Experiments (IMPROVE) guidelines.^57^ Before experimental procedures, the animals underwent a period of 10 days of adaptation to the researchers and the laboratory. Thereafter, animals were numbered and randomly assigned to sham or MCAO groups before surgery. During surgery, the animals received anesthesia with 2.5% isoflurane (RWD Life Science, Shenzhen, China) and suitable depth of anesthesia was confirmed by absence of the pedal withdrawal reflex. Body temperature, monitored by rectal probe, was maintained at 37.0 ± 0.05°C with a homeothermic blanket system (Harvard Apparatus, Holliston, MA, USA). Hair removal cream was applied to the area between the left ear and left eye. The site was washed repeatedly with 70% alcohol and iodine. The site was anesthetized topically with 0.5% bupivacaine (0.1 mL) before being incised. The temporal muscle was retracted to expose the temporal and parietal bones. Afterward, a 7/0 round surgical nylon monofilament was inserted into the left internal carotid artery via the external carotid artery stump until the nylon was inserted to approximately the marked location, that is, a depth of 1 cm. Following 60 min of MCAO, the filament was withdrawn to allow reperfusion. Animals in the sham group had the identical treatment, with the exception of blocking the middle cerebral artery. CBF was measured and recorded using a laser speckle imaging system (RFLSI III, RWD Life Science). A total of 309 C57BL/6JNifdc male mice were used for the study. *n*  =  5, 8, or 11 mice were utilized in each group. Seventeen mice were excluded because of death (seven mice), cerebral hemorrhage (four mice), or failure of ischemia induction (six mice) during the MCAO surgery. After stroke induction, the animals that met inclusion criteria were again randomized into three groups: an MCAO control group and two experimental groups (Tat‐Scr group and Tat‐CTM group). All animals had neurological deficits after MCAO surgery. After the second experimental animal grouping, no animals were died before the experimental end point 35 days and no animals were excluded from final analysis. Care for animals postoperatively included prevention of hypothermia by maintaining normal body temperature, intermittent oxygen exposure, administration of analgesics, infusing saline to maintain hydration, close‐monitoring postischemia, and liquid diet.

### 4.7 Neurological score

The neurological function of the experimental mice was evaluated blindly by independent researchers. Following MCAO, mNSS were used to assess neurological dysfunction. To determine the mNSS, a beam balance test (score of 0 to 6), reflex and abnormal movement test (score of 0–2) and motor test (including assessment of forelimb and hindlimb flexion as well as head movement, score of 0–6) were performed. Cumulative scores of 1–4, 5–9, and 10–14 represented minor, moderate, and severe damage, respectively.

### 4.8 Immunofluorescence staining

The cells were permeabilized with 0.5% Triton X‐100 for 15 min after being fixed in 4% buffered formaldehyde overnight. After receiving blocking with 10% donkey serum, they underwent an overnight incubation with the relevant primary antibodies. Then, they were incubated with Alexa 594‐conjugated antimouse and Alexa 488‐conjugated antirabbit antibodies (Jackson ImmunoResearch, West Grove, USA) at room temperature for 60 min and with 4,6‐diamidin‐2‐phenylindol (DAPI, 1:1000, Sigma‒Aldrich, Shanghai, China) at room temperature for 5 min. A fluorescence microscope (Olympus, BX53, Tokyo, Japan) was used to visualize the fluorescence signals. Regional fluorescence intensities were quantified using ImageJ software.

### 4.9 TUNEL staining

As we previously reported, TUNEL staining was carried out using a commercial kit (Roche, Rotkreuz, Switzerland) to assess cell apoptosis.^58^ Briefly, the slices were incubated with the TUNEL reaction mixture at 37°C for 60 min after fixation with 4% formaldehyde solution for 15 min at room temperature. TUNEL‐positive cells in a continuous 1 mm^2^ region of the ischemic penumbra were counted in five randomly selected mice from each group. To determine the average numbers of TUNEL‐positive cells per visual field in each mouse, an observer without knowledge of the research designs counted the numbers of TUNEL‐positive cells in each segment of 20 consecutive visual fields divided into four sections for each mouse.

### 4.10 Rotarod test

The rotarod test was used to assess the animals' motor coordination.^59^ Briefly, mice are kept on a rotation bar, and the rotor was accelerated (10 speeds from 4 to 40 rpm over 5 min). The time from the mouse being put on the rotating bar to dropping was recorded; a time of 300 s was recorded for mice that did not fall off the rod. To establish stable baseline values, all mice were trained three times per day for 2 days prior to surgery. The mean latency was recorded. After ischemic injury and peptide administration, the mice underwent the rotary test three times at the indicated time points.

### 4.11 Adhesive removal test

For analysis of somatosensory and motor impairment, mice were subjected to the adhesive test.^60^ In brief, a 3 ×3 (mm^2^) sticker was lightly attached to the palsied forepaw. Next, the mouse was returned to its cage, and we started the timer. The time from the beginning of the test to when the mouse touches the sticker was recorded as the touch time. The period of time from the start of the tests until the mice successfully removed the stickers was noted as the time of removal. Each animal underwent three trials each day on each front paw, with a 5‐min break between trials.

### 4.12 Cylinder test

By using the cylinder test, forepaw usage and rotation asymmetry were assessed. To evaluate sensorimotor function, the animal was positioned into a translucent plexiglass cylinder (15 cm high, 9 cm in diameter) surrounding a mirrored panel. The cylinder was recorded on video for 10 min. The number of forepaw touches was counted (left, L; right, R; both, B) for analysis, and the asymmetry rate was calculated as (L − R) / (L + R + B) × 100 (%).

### 4.13 Statistical analysis

Statistical analyses were performed using GraphPad Prism software (version 8.3.0, CA, USA). Data are expressed as mean ± SD from at least three independent experiments. The sample size (*n*) for each experiment was determined by preliminary tests and previous similar experiments and is indicated in the figure legends. Two‐tailed Student's t test was applied to assess the difference in mean between two groups. Differences in means among multiple groups were analyzed using one‐way or two‐way analysis of variance (ANOVA) then either Dunnett's post hoc test or Tukey's post hoc test. The escape latency in the training phase of the MWM tests (6 days) was assessed with repeated‐measures (RM) ANOVA. Discontinuous neurological deficit score data and plateau position crossover data from the day 7 exploratory trial were assessed with the Kruskal‒Wallis nonparametric test. All differences for which *P* < 0.05 were considered statistically significant (*P < 0.05, **P < 0.01, ***P < 0.001, and ****P < 0.0001; ns: not significant).

## AUTHOR CONTRIBUTIONS

Q.X., X.L., and Y.L.Z. acquired the funding, conceived, and designed the study. Q.X., X.Z., G.F.Z., L.Z., and M.M. performed the experiments and statistical analysis. Q.X. and X.L. created the figures. Y.Z. provided valuable advice. Q.X. and X.L. wrote and edited the manuscript. All authors have read and approved the final manuscript.

## CONFLICT OF INTEREST STATEMENT

The authors declare no competing interests.

## ETHICS STATEMENT

All experiments were approved by the Experimental Animal Care and Use Committee of Tongji Hospital, Tongji Medical College, Huazhong University of Science and Technology (No. TJH‐2021020036), and were performed in accordance with the National Institutes of Health Guidelines for the Care and Use of Laboratory Animals.

## Supporting information

Supporting Information

## Data Availability

All data needed to evaluate the conclusions in the paper are presented in the paper and/or the Supplementary Materials. Additional data related to this paper may be requested from the authors.
