# Supplementary material for: A cell‐penetrating peptide exerts therapeutic effects against ischemic stroke by mediating the lysosomal degradation of sirtuin 5
Source: MedComm (2020). 2023 Dec 13;4(6):e436. doi: 10.1002/mco2.436 (PMC10716672; doi:10.1002/mco2.436)
Supplement: Supplementary file 1 — Supporting Information [file MCO2-4-e436-s001.docx]

**A cell-penetrating peptide exerts therapeutic effects against ischemic stroke by mediating the lysosomal degradation of sirtuin 5**

**Running title: Tat-SIRT5-CTM peptide protects against stroke**

**Authors:** Qian Xia^1^, Xue Zhang^1^, Gaofeng Zhan^1^, Lu Zheng^2^, Meng Mao^3^, Yin Zhao^4^, Yilin Zhao^1^*, Xing Li^1^*

**Affiliations：**

^1^Department of Anesthesiology, Hubei Key Laboratory of Geriatric Anesthesia and Perioperative Brain Health, and Wuhan Clinical Research Center for Geriatric Anesthesia, Tongji Hospital, Tongji Medical College, Huazhong University of Science and Technology, Wuhan 430030, China

^2^Department of Transfusion, The First Affiliated Hospital of Zhengzhou University, Zhengzhou 450000, China

^3^Department of Anesthesiology and Perioperative Medicine, Zhengzhou Central Hospital Affiliated to Zhengzhou University, Zhengzhou 450007, China

^4^Department of Ophthalmology, Tongji Hospital, Tongji Medical College, Huazhong University of Science and Technology, Wuhan 430030, China.

***Corresponding authors:**

Xing Li, Department of Anesthesiology, Tongji Hospital, Tongji Medical College, Huazhong University of Science and Technology, 1095 JieFang Avenue, Wuhan 430030, China

**E-mail:** lixing88@hust.edu.cn;

Yilin Zhao, Department of Anesthesiology, Tongji Hospital, Tongji Medical College, Huazhong University of Science and Technology, 1095 JieFang Avenue, Wuhan 430030, China

**E-mail:** yilinzhao@hust.edu.cn

**Supplementary Figures and Legends**

**Supplementary Figure 1**

**
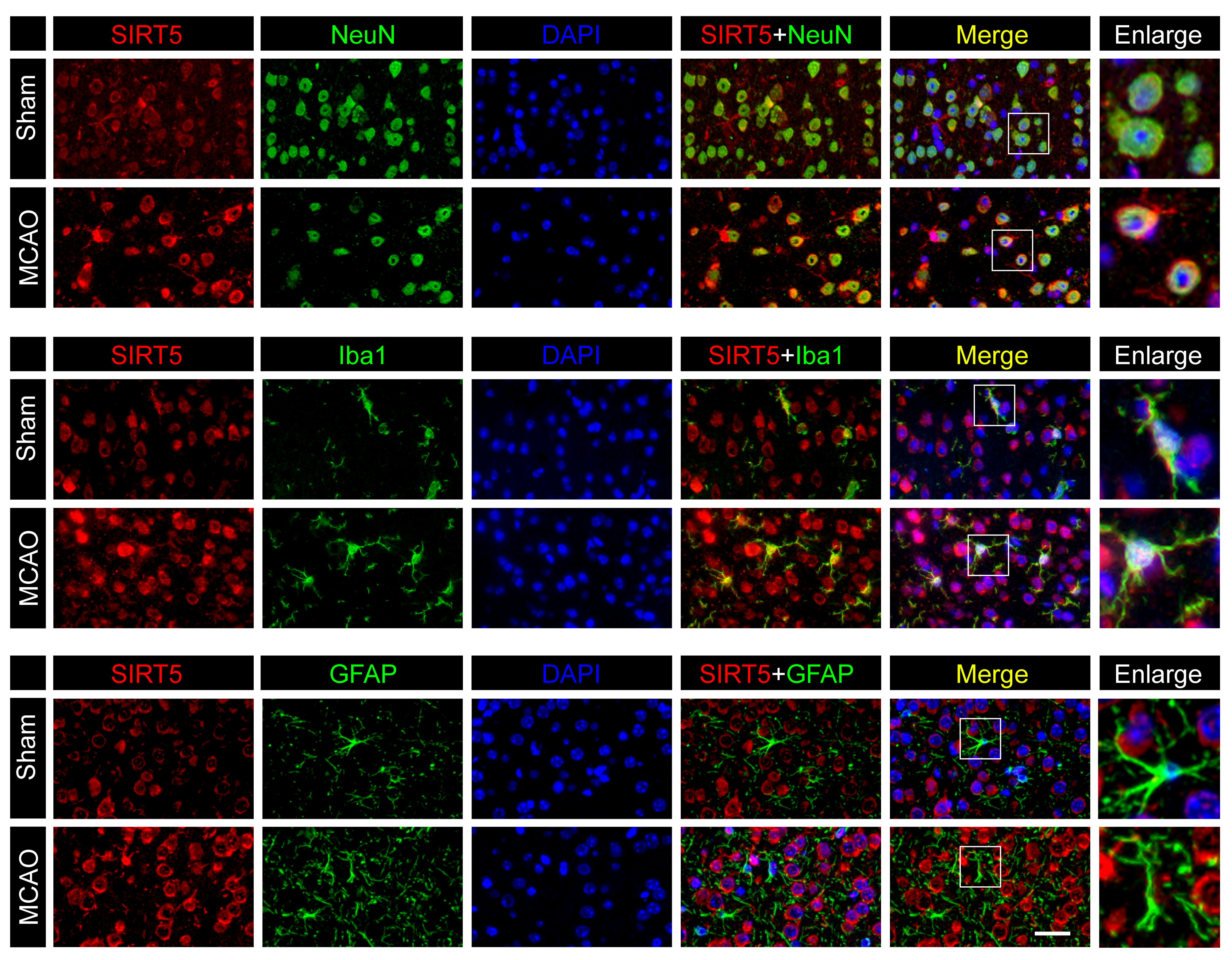
**

**Figure S1.** Representative double immunostaining of SIRT5 (red) with NeuN (a neuronal marker, green), Iba1 (a microglial marker, green) and GFAP (an astrocyte glial marker, green) from ischemic penumbra of brain tissue after MCAO operation. n = 5 mice per group, scale bars: 20 μm.

**Supplementary Figure 2**

**
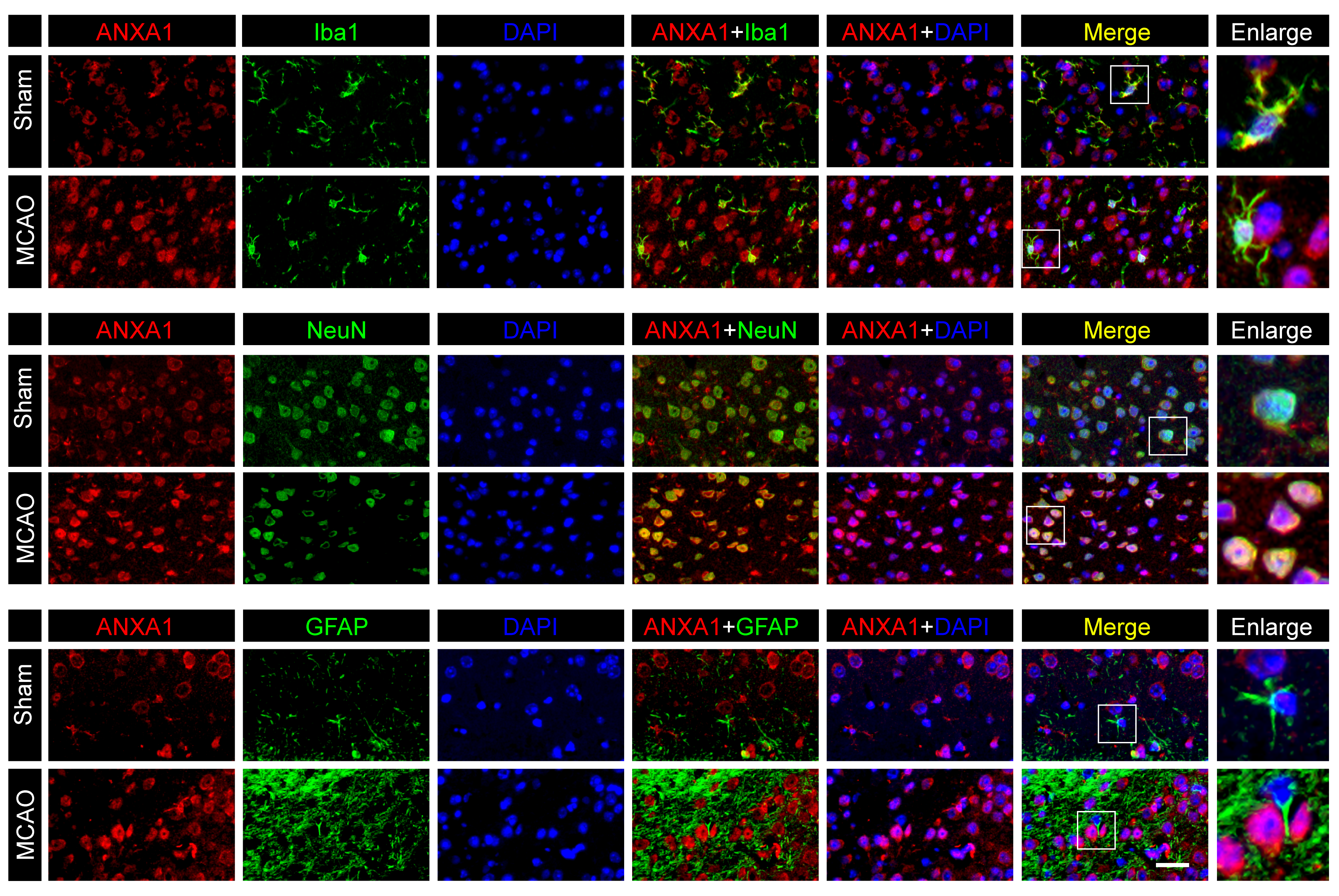
**

**Figure S2.** Representative double immunostaining of ANXA1 (red) with Iba1 (a microglial marker, green), NeuN (a neuronal marker, green) and GFAP (an astrocyte glial marker, green) from ischemic penumbra of brain tissue after MCAO operation. n = 5 mice per group, scale bars: 20 μm.

**Supplementary Figure 3**

**
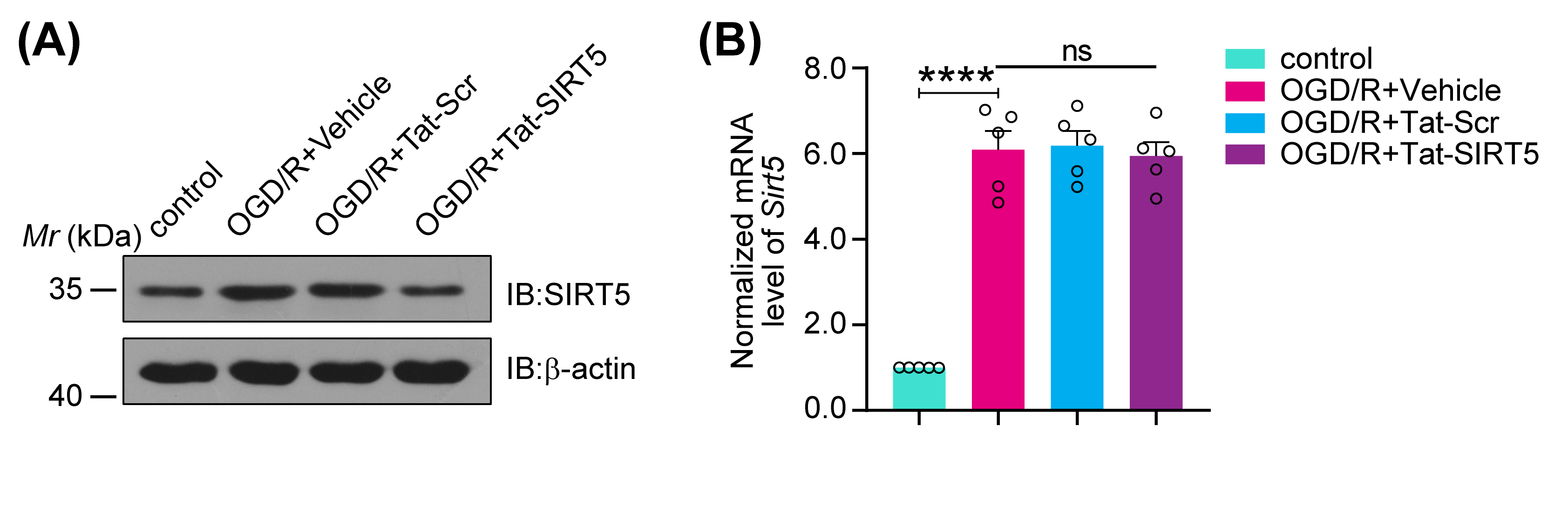
**

**Figure S3.** Primary cultured neurons were treated with vehicle, Tat-SIRT5-CTM or Tat-Scr peptide and then subjected to OGD/R. (A) Representative immunoblot showing the protein expression of SIRT5 in primary neurons. (B) RT-qPCR shows the mRNA levels of *Sirt5* in primary neurons. The data are expressed as the mean ± SD from five independent experiments. *Mr*, relative molecular mass. *****P* < 0.0001. ns: no significance.

**Supplementary Figure 4**

**
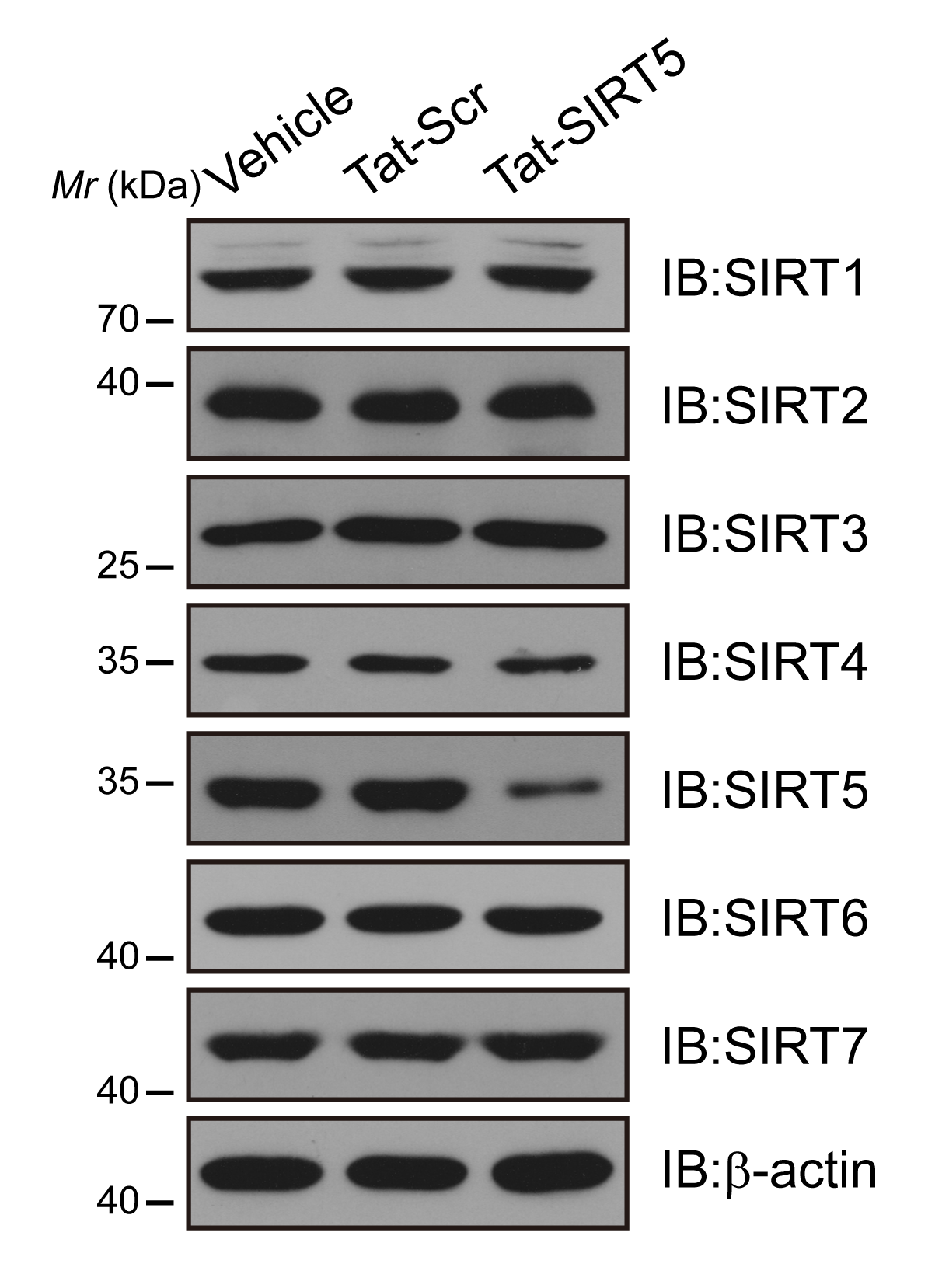
**

**Figure S4. The** **Tat-SIRT5-CTM peptide specificity promoted the degradation of SIRT5.** Representative immunoblot analysis showing the protein level of SIRT1-7 in primary cultured microglia treated with Tat-SIRT5-CTM. Data represented of three independent experiments.

**Supplementary Figure 5**

**
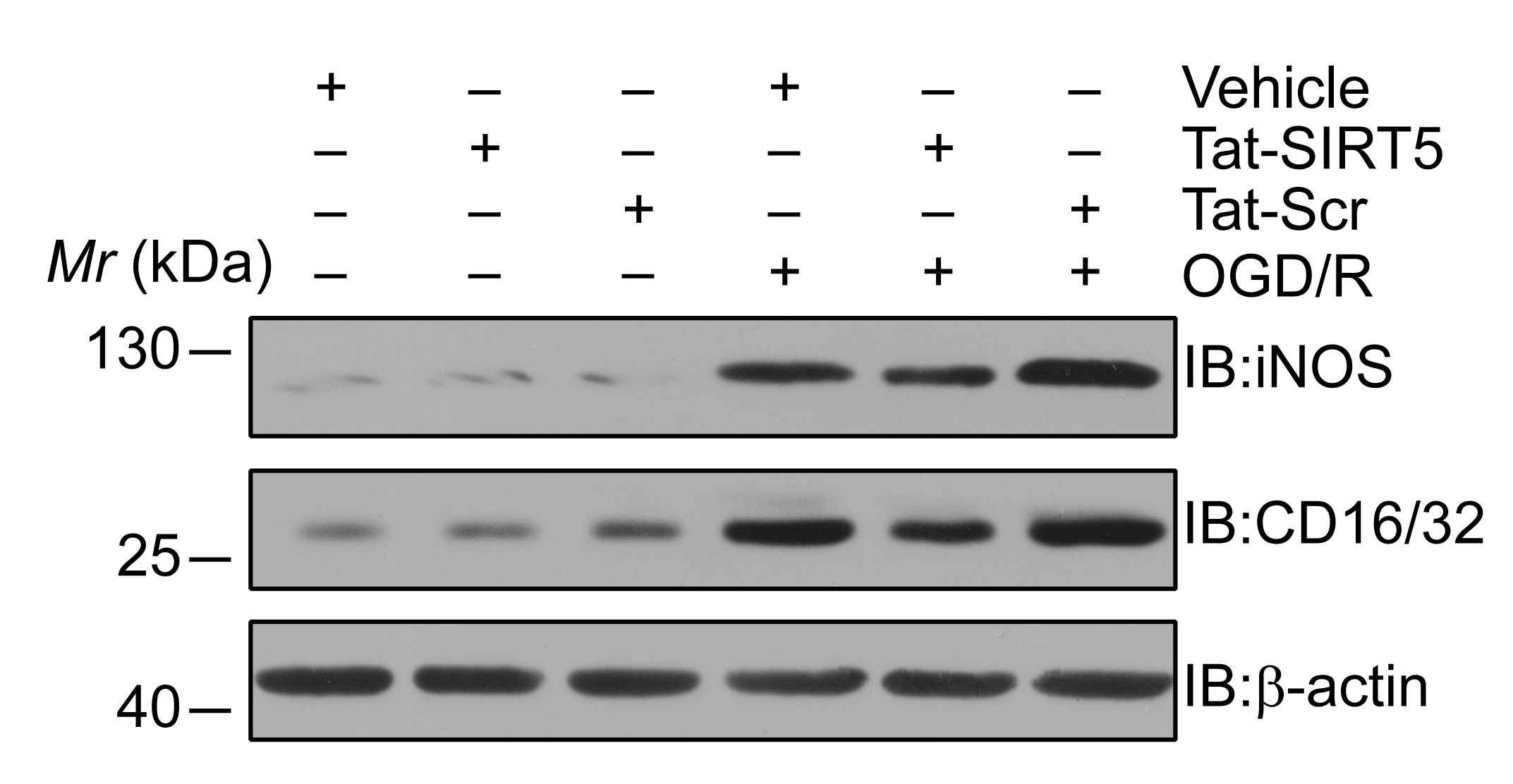
**

**Figure S5.** Tat-SIRT5-CTM treatment decreased the expression of the proinflammatory mediators iNOS and CD16/32 in microglia following OGD/R. Data represent five independent experiments.

**Supplementary Figure 6**

**
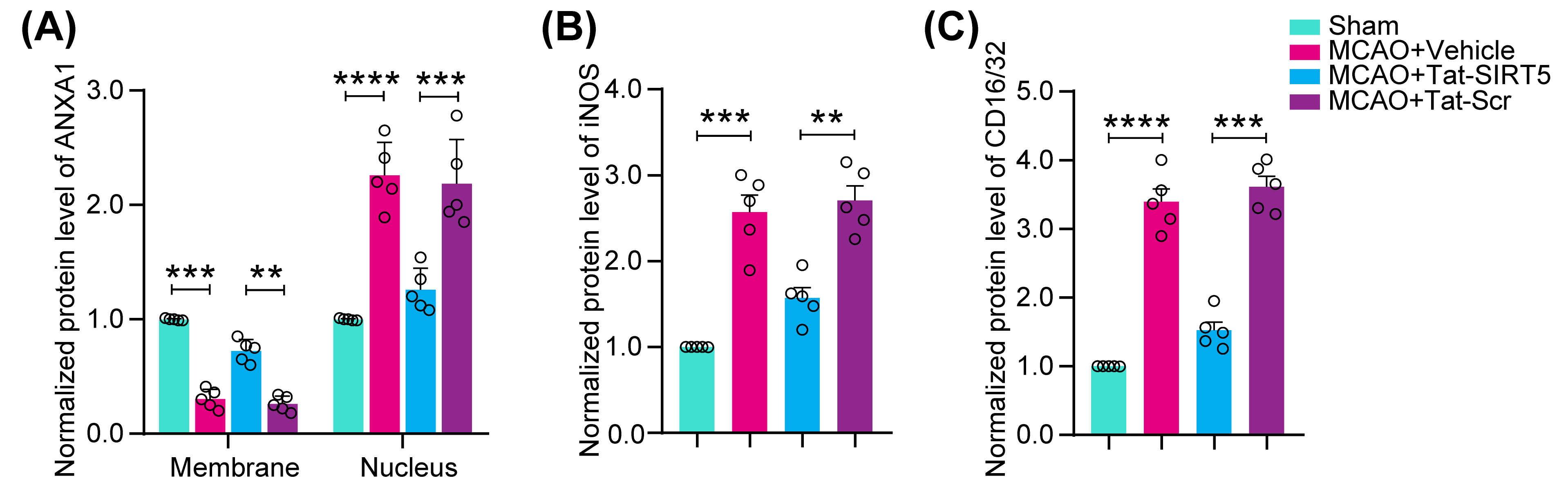
**

**Figure S6. Quantification analysis of the data shown in Figures 5F and G.** (A) Statistical analysis of the data shown in Figure 5F. (B, C) Statistical analysis of the data shown in Figure 5G. Data are expressed as the mean ± SD. n = 5 mice per group. ***P* < 0.01, ****P* < 0.001, and *****P* < 0.0001.

**Supplementary Figure 7**

**
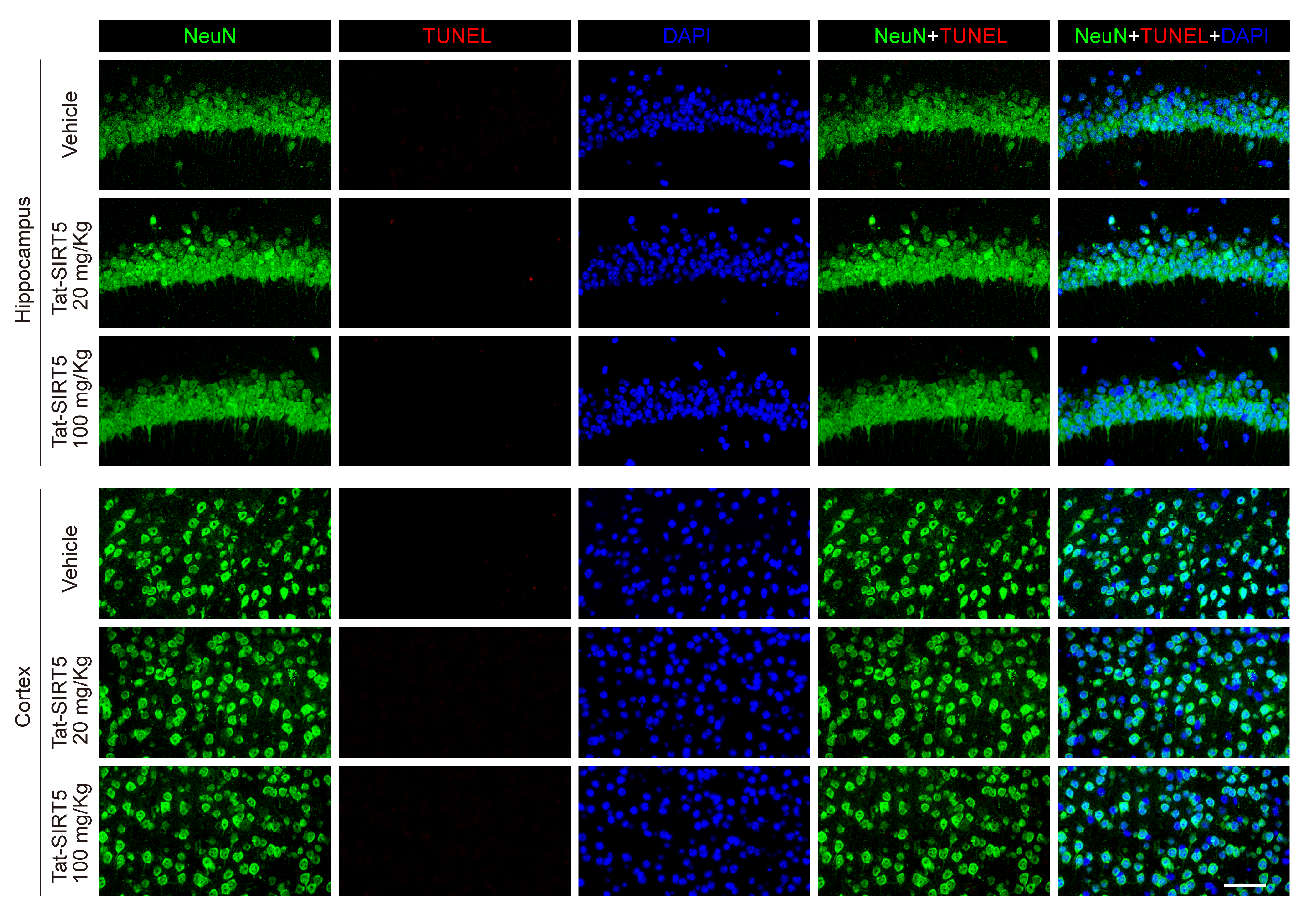
**

**Figure S7. The Tat-SIRT5-CTM peptide treatment does not affect neuronal survival in nonischemic animals.** Mice were administrated with Tat-SIRT5-CTM (i.v.) at a concentration of 20 mg/kg or 100 mg/kg daily for continuous 7 days. Representative images showing the TUNEL positive cells in the hippocampus and cortex of mice (n = 6 mice per group). Scale bar = 50 μm.

**Supplementary Figure 8**


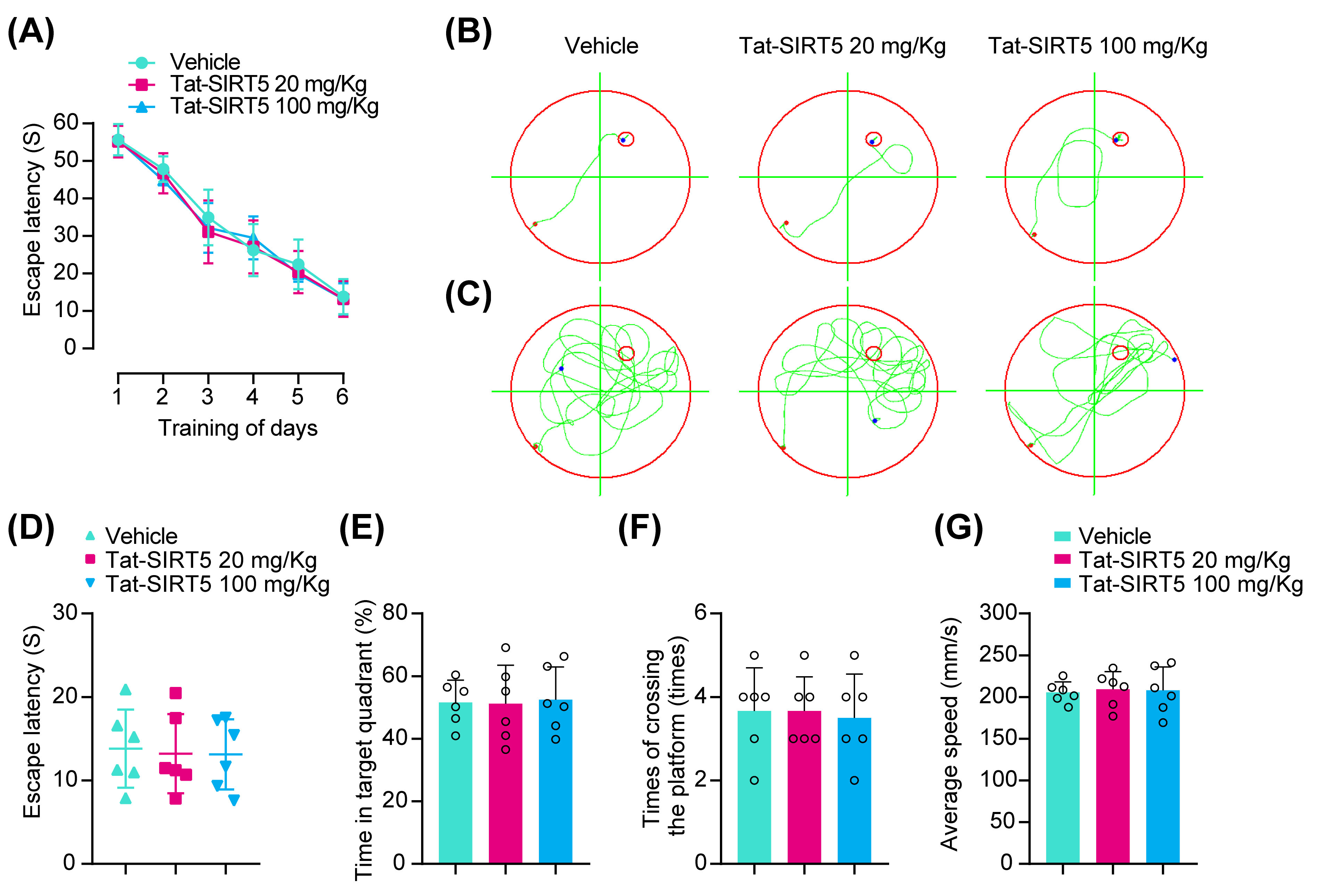


**Figure S8. The Tat-SIRT5-CTM peptide does not affect cognitive function in nonischemic animals.** Mice were administrated with Tat-SIRT5-CTM (i.v.) at a concentration of 20 mg/kg or 100 mg/kg daily for continuous 7 days. Latency trial (A, B) and probe trial (C-F) results in the MWM for the animals. (A) Escape latency to find the platform on days 1-6 d after Tat-SIRT5-CTM administration. (B, C) Representative traces indicating the paths of the mice during the maze latency trials (B) and the swimming traces from probe trials (C). (D) Exploration time spent finding the submerged platform at 6 d following reperfusion during the latency trial. (E) Time spent in the safety quadrant during the probe trial. (F) Number of times the mice crossed the platform location on day 7 (probe trial) and the swimming speed (G). The data are expressed as the mean ± SD. n = 6 mice per group.

**Supplementary Figure 9**


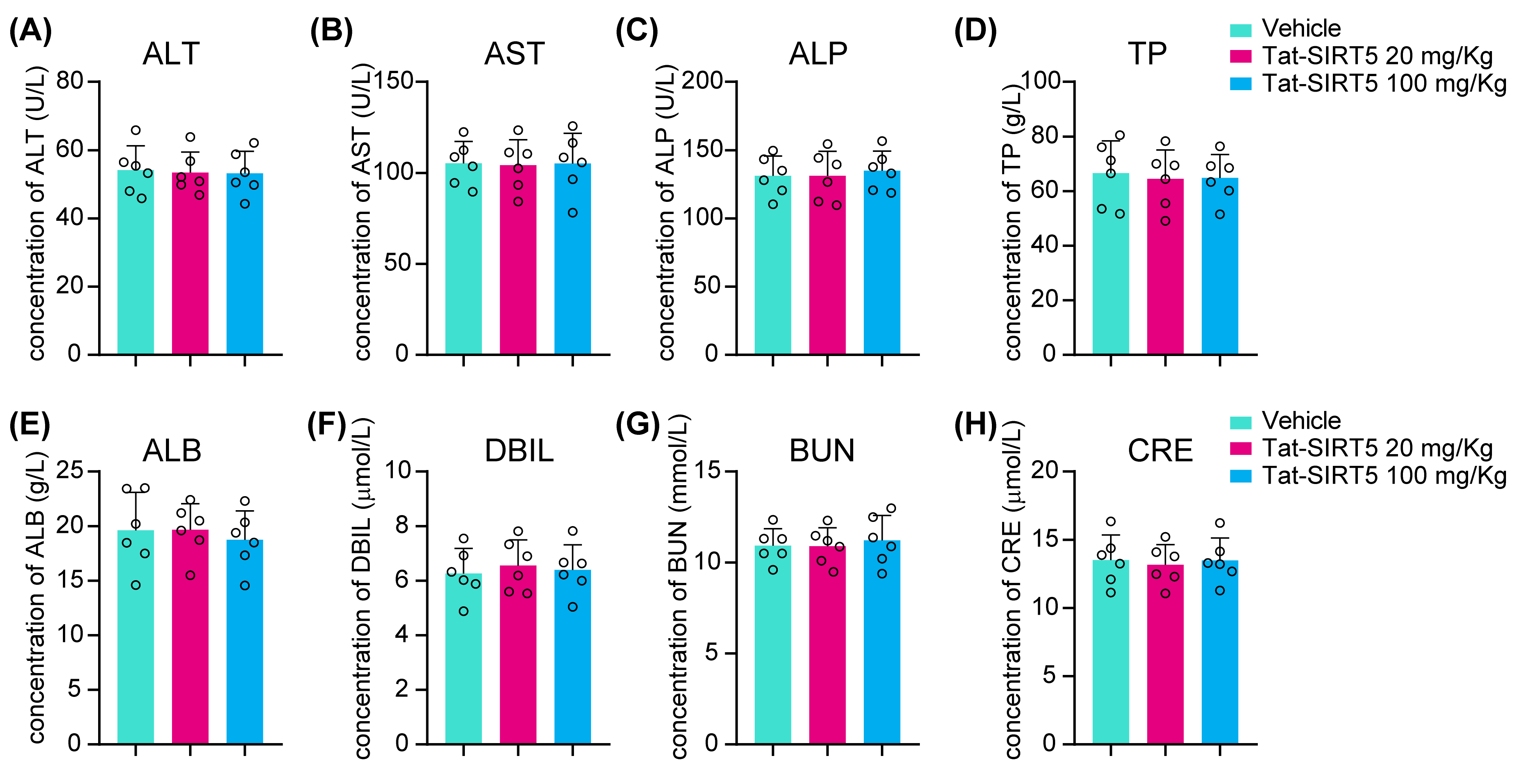


**Figure S9. Safety profile of Tat-SIRT5-CTM.** Assays of ALT, AST, ALP, TP, ALB, DBIL, BUN, and CRE levels in the plasma of mice treated with Tat-SIRT5-CTM (i.v.) at a concentration of 20 mg/kg or 100 mg/kg daily for continuous 7 days. ALT, alanine aminotransferase; AST, aspartate aminotransferase; ALP, alkaline phosphatase; TP, total protein; ALB, albumin; DBIL, direct bilirubin; BUN, blood urea nitrogen, and CRE, creatinine. The data are expressed as the mean ± SD. n = 6 mice per group.

**Supplementary Figure 10**


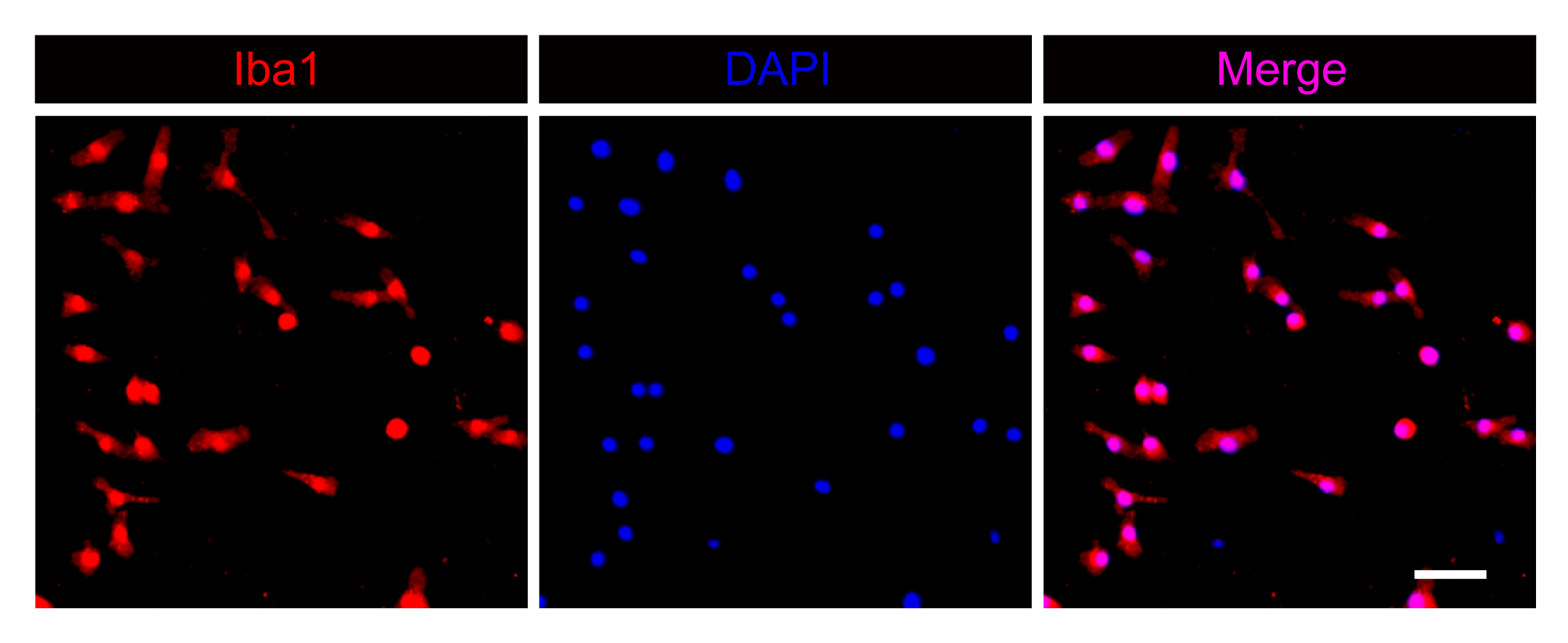


**Figure S10. Immunofluorescence analysis shows the purity of primary cultured microglial cells.** Cells were fixed and stained for microglia specific marker Iba1 (red). Scale bar = 50 μm.

**Table S1. Primers used in this study.**

| Primer name | Primer sequences (5’- 3’) | |
| --- | --- | --- |
|  | Forward | Reverse |
| Quantitative RT-PCR primers | | |
| *Sirt5* | GCCACCGACAGATTCAGGTT | CCACAGGGCGGTTAAGAAGT |
| *Il-1β* | GAAAGACGGCACACCCAC | TGTGACCCTGAGCGACCT |
| *Il-6* | TCTCTGGGAAATCGTGGAA | GATGGTCTTGGTCCTTAGCC |
| *Tnf-α* | ACGGCATGGATCTCAAAGAC | AGATAGCAAATCGGCTGACG |
| *Cxcl1* | GAGCTTGAAGGTGTTGCCCT | CGCGACCATTCTTGAGTGTG |
| *Ccl2* | GCAGGTCCCTGTCATGCTTC | GTGGGGCGTTAACTGCATCT |
| *β-actin* | TTCGTTGCCGGTCCACACCC | GCTTTGCACATGCCGGAGCC |

**Supplementary Materials and Methods**

**Cell culture**

Primary neurons were dissected from embryonic (E16–E18) mice. Briefly, after dissecting and cutting the cerebral cortex under an anatomic microscope, we digested the neurons with 0.25% trypsin–EDTA (Sigma‒Aldrich, St. Louis, MO, USA) solution for dissociation. Next, the cells were centrifuged, and the cell suspensions were collected. A 24-well culture plate with 10% fetal bovine serum (FBS, Gibco, Gaithersburg, MD, USA) and Dulbecco's modified Eagle's minimum essential medium (DMEM, Thermo Fisher Scientific, Waltham, MA, USA) was used to seed the cells after they had been counted. After 24 hours, the cultures were replaced with Neurobasal medium (Gibco, Gaithersburg, MD, USA) supplemented with 2% B-27 (Gibco) and fed twice a week. For the primary microglia, after two weeks of culture in vitro, the microglia matured. Using a horizontal shaker at 37.0 °C and 200 rpm for 6 hours, we separated mixed glial cells. Finally, the microglial supernatant was collected and cultured in 6-well or 24-well culture plates, and the medium was replaced every 3 days. The purity of adherent cells was verified by immunofluorescence staining, which indicated more than 95% of the cells in cultures were positive for the microglia-specific marker Iba-1 (ab283319, Abcam, Boston, MA, USA) (Figure S8).

**Protein extraction and preparation, and western blotting**

Brain tissues and cultured cells were lysed in RIPA buffer (Beyotime Biotechnology, Shanghai, China) supplemented with cOmplete™ protease inhibitor cocktail tablets (5 mg/ml; Roche Diagnostics, Basel, Switzerland) at 4 °C for 15 min. The extract was then centrifuged at 14,000 × g for an additional 15 min, and the supernatant was gathered and employed. Equal amounts of protein extracts were electrophoresed on 10% or 12% sodium dodecyl sulfate–polyacrylamide gel electrophoresis (SDS‒PAGE) before being transferred to polyvinylidene fluoride (PVDF) membranes (Roche, Basel, Switzerland). The membranes were treated at 4 °C overnight with primary antibodies after being blocked with 5% BSA. The membranes were then treated with a secondary antibody (1:20,000; Jackson ImmunoResearch, West Grove, USA) and incubated for 1 hour. Finally, immunodetection was carried out using a chemiluminescence substrate kit (Thermo Pierce, Rockford, USA).

**Co-IP**

Detection of the succinylation level of ANXA1 were performed by co-IP with antibodies against ANXA1 followed by immunoblot analysis with antibodies against Succ-K and ANXA1. Briefly, fresh samples were gathered, and the BCA protein concentration measurement kit (Beyotime Biotechnology, Shanghai, China) was used to determine the presence of proteins, and then the supernatants were treated with anti-ANXA1 antibody overnight at 4 °C. The samples were mixed with Protein A/G Plus-agarose beads (Beyotime Biotechnology) and incubated for 2 hours at room temperature. Following three washes with 0.01 mol/L PBS, the samples were boiled, mixed with 2 × loading buffer, and run on SDS‒PAGE before being transferred to PVDF membranes for immunoblotting. For measurement of the amount of input, aliquots of the original lysates were also run in parallel on SDS‒PAGE for immunoblotting. The band intensities of western blotting were analyzed by ImageJ software (NIH, Baltimore, MD, USA) with background subtraction.

**RNA extraction and qRT-PCR**

Following the manufacturer's instructions, total RNA from primary cultures was extracted using TRIzol reagent (Invitrogen, Carlsbad, CA, USA). With the ReverTra Ace-TM First Strand cDNA Synthesis Kit (Toyobo, Osaka, Japan), the reverse transcription procedure was carried out. Five nanograms of cDNA and 300 nM of the particular oligonucleotides listed in (Table S1) were used in the qRT-PCR experiments. An Applied Biosystems StepOnePlusTM Real-Time PCR System (Foster City, CA, USA) was employed. The relative mRNA expression values were standardized to *β-actin* when analyzing gene expression using the 2^−ΔΔCt^ method.

**ELISA**

ANXA1 release was measured using an ELISA kit purchased from Cloud-Clone Corp (SEE787Mu, Wuhan, China), and proinflammatory cytokine and chemokine IL-1β, IL-6, TNF-α, CXCL1, and CCL2 expression in the supernatant of cell culture medium and brain tissues was determined with a mouse enzyme-linked immunosorbent assay (ELISA) kit (R&D Systems, Minneapolis, MN, USA) according to the manufacturer’s instructions. Standards and samples were each run three times. Samples were taken in tubes devoid of pyrogen and endotoxins. An EnspireTM multilabel reader 2300 was used to detect absorbance at 450 nm (PerkinElmer, Waltham, MA, USA).

**Peptide dose–effect experiments**

The vehicle/Tat-SIRT5-CTM/Tat-Scr-CTM peptide was intravenously injected into mice undergoing a 60-min MCAO at a single dose of 1, 5, 10, 20 or 50 mg/kg body weight. The cortical proteins were extracted, and all the mice were euthanized 24 hours later. The proteins were precipitated using anti-ANXA1 antibodies and blotted with anti-SIRT5 antibodies. There was no statistically significant difference in the ratio of precipitated ANXA1 between the treatments. Subsequent studies will employ a dose of 20 mg/kg.

**MWM test**

The spatial learning and memory of animals were detected using the MWM test. In summary, the water maze was made up of a circular tank with a diameter of 120 cm and a height of 60 cm filled with opaque water, as well as a round platform with a diameter of 6 cm that was submerged 1 cm under the water's surface at a temperature of 22° ± 2 °C. The tank's walls were painted with various shapes that acted as spatial cues. The swimming traces in the water maze were recorded using a computerized tracking device set above the maze (Xinruan Information Technology, Shanghai, China). Mice spent 30 min getting used to the testing environment before testing. Each experimental animal underwent a 2-day adaptation phase in the behavior room, which was kept at 25 °C (a residential space during training). The training phase was seven days long. Titanium dioxide was added to water to make it opaque for C57BL/6JNifdc mice. To encourage learning and directional memory, we allowed the mice 60 s to locate the platform and permitted them to stay there for 15 s. The mice were placed from four separate random release points during the first six days of four tests.

**TTC Staining**

After neurological functional assessment, mice were sacrificed under deep anesthesia. The mouse brains were dissected, sliced into six 2-mm-thick coronal sections with a matrix (RWD Life Science, Shenzhen, China). After that, the sections were submerged in 1% TTC (Sigma‒Aldrich) at 37 °C for 30 min. Slices were rotated and rinsed three times with ddH_2_O once every 5 min. After the portions were fixed in 4% paraformaldehyde, they were then imaged using a digital camera. The infarct areas (white) were measured by an observer blinded to the experimental groups using ImageJ software. Considering the thickness of the sections, the corresponding volumes were calculated and corrected for the effects of edema, estimated by comparing the total volumes of the cerebral hemispheres. Corrected infarct volumes are expressed as a percentage relative to the contralateral hemisphere to correct for normal size differences between animals.
